# Pharmacoinformatics and UPLC-QTOF/ESI-MS-Based Phytochemical Screening of *Combretum indicum* against Oxidative Stress and Alloxan-Induced Diabetes in Long–Evans Rats

**DOI:** 10.3390/molecules26154634

**Published:** 2021-07-30

**Authors:** Md. Shaekh Forid, Md. Atiar Rahman, Mohd Fadhlizil Fasihi Mohd Aluwi, Md. Nazim Uddin, Tapashi Ghosh Roy, Milon Chandra Mohanta, AKM Moyeenul Huq, Zainul Amiruddin Zakaria

**Affiliations:** 1 Department of Pharmacy, School of Science and Engineering, Southeast University, Dhaka 1213, Bangladesh; foridpharmacy91@gmail.com; 2 Department of Biochemistry & Molecular Biology, University of Chittagong, Chittagong 4331, Bangladesh; 3 Faculty of Industrial Science and Technology, Universiti Malaysia Pahang, Kuantan 26300, Pahang, Malaysia; fasihi@ump.edu.my; 4 Institute of Food Science and Technology, Bangladesh Council of Scientific and Industrial Research, Dhaka 1205, Bangladesh; nazimbio@yahoo.com; 5 Department of Chemistry, University of Chittagong, Chittagong 4331, Bangladesh; tapashir57@cu.ac.bd; 6 Department of Chemistry, School of Science and Engineering, Tulane University, New Orleans, LA 70118, USA; milonmohanta@yahoo.com; 7 Department of Pharmacy, School of Medicine, University of Asia Pacific, 74/A, Green Road, Dhaka 1205, Bangladesh; 8 Department of Biomedical Science, Faculty of Medicine and Health Sciences, Universiti Putra Malaysia, UPM Serdang, Serdang 43400, Selangor, Malaysia

**Keywords:** *Combretum indicum* L., antidiabetic activity, histopathology, UPLC-QTOF/ESI-MS, network pharmacology

## Abstract

This research investigated a UPLC-QTOF/ESI-MS-based phytochemical profiling of *Combretum indicum* leaf extract (CILEx), and explored its in vitro antioxidant and in vivo antidiabetic effects in a Long–Evans rat model. After a one-week intervention, the animals’ blood glucose, lipid profile, and pancreatic architectures were evaluated. UPLC-QTOF/ESI-MS fragmentation of CILEx and its eight docking-guided compounds were further dissected to evaluate their roles using bioinformatics-based network pharmacological tools. Results showed a very promising antioxidative effect of CILEx. Both doses of CILEx were found to significantly (*p* < 0.05) reduce blood glucose, low-density lipoprotein (LDL), and total cholesterol (TC), and increase high-density lipoprotein (HDL). Pancreatic tissue architectures were much improved compared to the diabetic control group. A computational approach revealed that schizonepetoside E, melianol, leucodelphinidin, and arbutin were highly suitable for further therapeutic assessment. Arbutin, in a Gene Ontology and PPI network study, evolved as the most prospective constituent for 203 target proteins of 48 KEGG pathways regulating immune modulation and insulin secretion to control diabetes. The fragmentation mechanisms of the compounds are consistent with the obtained effects for CILEx. Results show that the natural compounds from CILEx could exert potential antidiabetic effects through in vivo and computational study.

## 1. Introduction

Diabetes mellitus (DM) is a major public health concern associated with many debilitating health conditions. It is a complex and chronic metabolic disease in which the body’s ability to produce or respond to insulin is impaired. It was reported that about 451 million people aged 18–99 years were afflicted by diabetes in 2017, with 5 million deaths recorded worldwide. The prevalence is expected to rise to 693 million by 2045 [1]. The long-term symptoms of diabetes include vascular disorders such as retinopathy, nephropathy, neuropathy, cardiomyopathy, liver dysfunction, and dystrophy in skeletal muscle and adipose tissue [2,3]. The commonly available therapeutic agents, including sulfonylurea and biguanides, are not devoid of side effects, such as vomiting, anorexia, skin rashes, heartburn, and gastrointestinal discomfort [4,5]. Moreover, the cost of modern antidiabetic agents is also a big concern for patients, especially in underdeveloped and developing countries. Traditional medicines—predominantly herbal therapies—are attracting more interest worldwide to treat a wide range of ailments, including diabetes. Many plants have already been reported to be useful in the management of diabetes.

*Combretum indicum* L. (Combretaceae family), commonly known as Rangoon creeper, is an impressive tropical vine for outdoor gardens. It is distributed all over the world, especially in India, Sri Lanka, Nepal, Bangladesh, South China, Myanmar, and along the East Asian countries [6,7]. Traditionally, the plant is used for cough relief [8]. Its fruits and seeds are used as a vermifuge and for rickets in the Philippines, Thailand, and the Indochina region [9]. In Indonesia, the flowers are used in salads to add color [10]. In Bangladesh, its seeds are used for diarrhea, fever, boil, ulcers, and helminthiasis. Four diphenylpropanoids can be isolated from the stem bark [11], and the presence of linoleic, oleic, palmitic, and stearic acids is also reported [12].

A number of pharmacological studies—such as immunomodulatory, antimicrobial, antioxidant, antipyretic, anthelmintic, antirheumatic, antiviral, antifungal, antiseptic, antidiarrheal, and anti-hyperlipidemic studies—have also been reported [11,13,14,15,16,17,18,19,20,21,22,23]. In addition, the flower showed a hypoglycemic effect in rats [24]. However, there is no study available for its effect on diabetes-associated abnormal lipid profiles. Therefore, the current investigation was carried out to evaluate the antidiabetic and anti-hyperlipidemic effects of ethanol extracts of *C. indica* leaves (CILEx) on an alloxan-induced diabetic Long–Evans rat model.

## 2. Results

### 2.1. Determination of Total Phenolic and Total Flavonoid Contents

**Figure 1:**
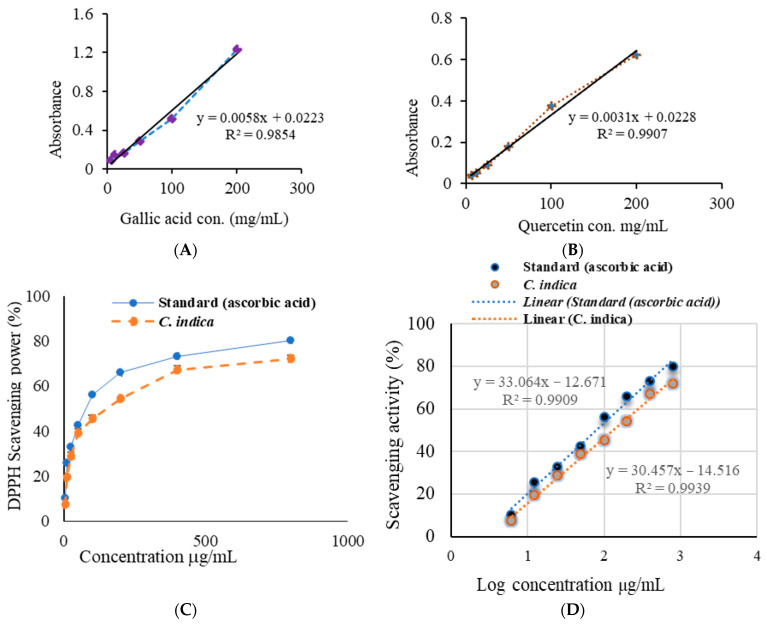
Antioxidative potential of CILEx: (**A**) total phenolic content, and (**B**) total flavonoid content calculated from the standard curve extrapolated against gallic acid and quercetin, respectively, used as standards; (**C**) 1,1-diphenyl-2-picrylhydrazyl (DPPH) free radical scavenging effect measured using ascorbic acid as a standard; (**D**) extrapolation of inhibition concentration (IC_50_) using the linear curve as a standard, and sampling by *t*-test.

The total phenolic and total flavonoid contents of CILEx are presented in Table 1. Total phenolic content was expressed by the GAE equivalent per gram of extract, which was determined as 155 ± 7.35 mg/g dry weight. The regression equation of the calibration curve (y = 0.005x + 0.022; r2 = 0.985) is shown in Figure 1A. The total flavonoid content of CILEx was 164.33 ± 2.71 mg/g dry weight expressed by quercetin (QE) equivalent per gram of extract. The regression equation of the calibration curve (y = 0.003x + 0.022; r2 = 0.990) is shown in Figure 1B.

### 2.2. DPPH Scavenging Activity

The results of DPPH free radical scavenging activity are presented in Figure 1C. The mean percentage scavenging activity of standard ascorbic acid (78.1 ± 0.63%) was significantly different from CILEx (72.9 ± 0.68%). The half-maximal inhibitory concentration (IC_50_) of the extract was 162.6 ± 3.10 µg/mL, which is significantly different (*p* < 0.05) from that of ascorbic acid (80.65 ± 2.8 µg/mL) (Figure 1D). As the cutoff value of radical scavenging activity is 1000 µg/mL, it is clear that the extract possess a high antioxidant potential. An IC_50_ value of any substance for this activity higher than the cutoff limit is considered to be ineffective.

### 2.3. Acute Oral Toxicity Study and Selection of Dose

In an acute toxicity study, the oral administration of CILEx in rats did not show any change in their behavioral patterns. No toxic effects were observed at the higher dose of 1000 mg/kg body weight. Therefore, CILEx at a dose of 1000 mg/kg was considered to be safer for administration in biological systems.

### 2.4. Effect of CILEx on Blood Glucose Levels

**Figure 2:**
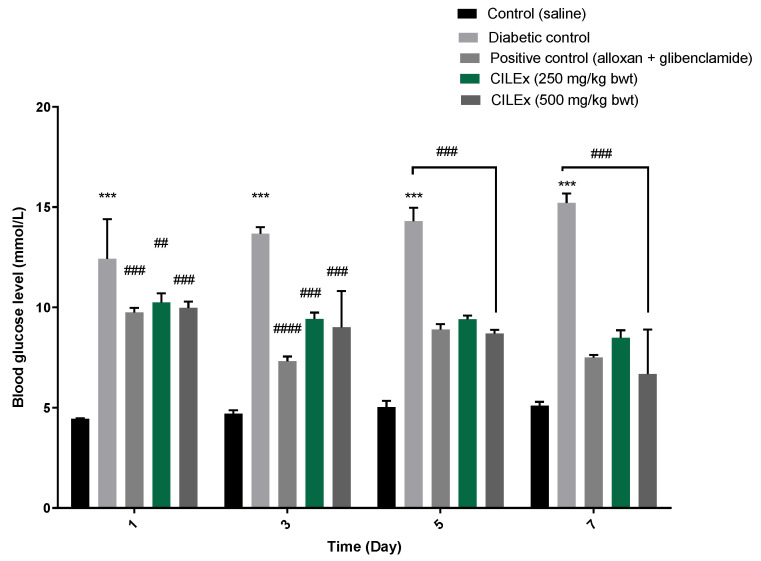
Effect of CILEx on blood glucose levels after a 7-day treatment. Glibenclamide was used as a positive control. The values are represented as mean ± SEM; n = 4, significant difference (*p* < 0.05) was calculated as compared to the untreated diabetic control group. In the figure, (***) over the bar denotes significant different from control, and (##, ###, ####) indicates significant difference from diabetic control.

The changes in blood sugar levels over one week are shown in Figure 2. The introduction of alloxan drastically increased the blood glucose level, which was highly significant (*p* < 0.001) compared to the normal control group, and remained constant throughout the study period. The administration of CILEx was able to reduce the elevated blood glucose level significantly (*p* < 0.001), compared to the diabetic control group, after every dosing with 250 and 500 mg/kg body weight A gradual blood glucose lowering effect was observed as the intervention continued for 7 days.

### 2.5. Effect of CILEx on Lipid Profiles in Animal Intervention

**Figure 3:**
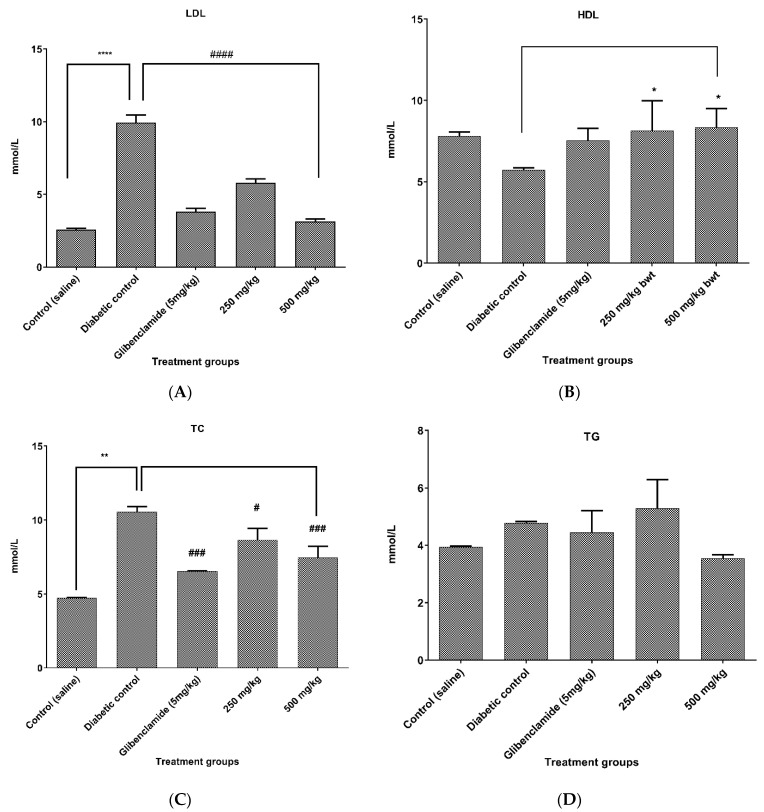
Effect of CILEx on serum lipid profiles: (**A**) low-density lipoprotein (LDL), (**B**) high-density lipoprotein (HDL), (**C**) total cholesterol (TC), and (**D**) triglyceride levels of different treatment groups. Glibenclamide (5 mg/kg body weight) was used as a positive control. Data were represented as mean ± SD of four animals, and were analyzed via one-way ANOVA using Tukey’s multiple range post hoc test. (*, **, ****) over the bar denotes significant different from control, and (#, ###, ####) indicates significant difference from diabetic control *p* < 0.05 was considered to be significant.

The serum lipid profiles of all of the groups were measured, as shown in Figure 3. Both of the doses of CILEx (250 and 500 mg/kg body weight) significantly decreased the LDL levels, to 5.8 ± 0.2 and 3.1 ± 0.2 mmol/L, respectively (*p* < 0.001), while HDL was simultaneously increased compared to the diabetic control group (8.1 ± 1.6 and 8.3 ± 1.0 mmol/L, respectively, *p* < 0.05). Reduced total blood cholesterol levels were found to be achieved, with 8.6 ± 0.7 mmol/L (*p* < 0.05) by 250 mg/kg, and 7.5 ± 0.7 mmol/L (*p* < 0.001) by 500 mg/kg. Triglyceride levels were also found to be lowered, but the change was statistically insignificant.

### 2.6. Effect of CILEx on Animals’ Tissue Architecture

**Figure 4:**
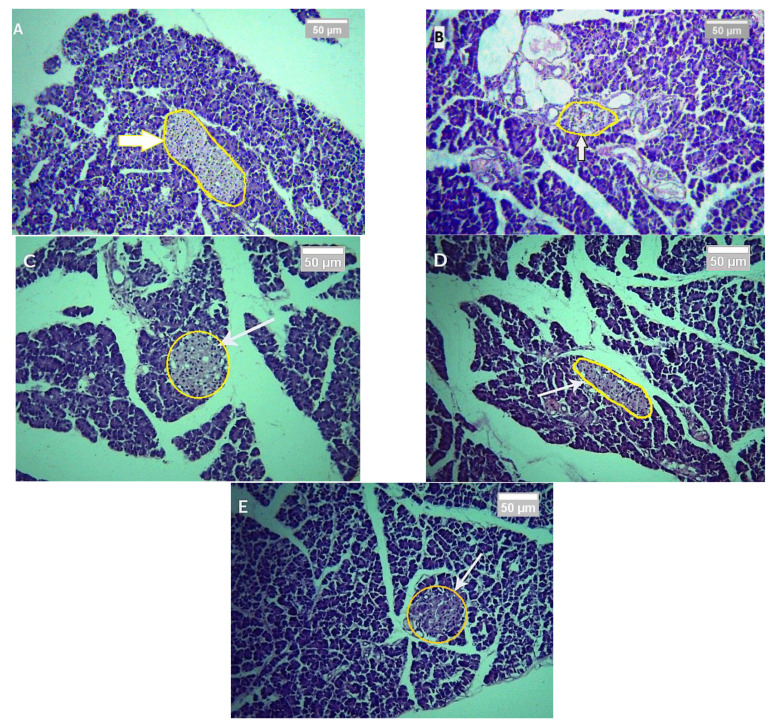
Histopathological slides of rat pancreas cells after 7 days of treatment: (**A**) normal control (presence of normal islet cells), (**B**) diabetic control (shrinkage of the islet cells), (**C**) diabetic + glibenclamide 150 mg/kg (increased number and size of islet cells), (**D**) diabetic + CILEx 250 mg/kg (improved size and shape of islet cells), and (**E**) diabetic + CILEx 500 mg/kg (restored number and size of islets).

The morphology of pancreatic tissues of different groups was examined through the hematoxylin and eosin staining method. The histopathological slides of pancreatic islets are shown in Figure 4A–E. It can be seen from the slides that group I, with normal rats, showed normal histological characteristics and islet structures. The group II alloxan-induced (150 mg/kg) rats were found to have a reduced number and size of the islet cells, thus causing shrinkage. The animals in groups IV and V—with diabetes, and treated with CILEx (250 mg/kg and 500 mg/kg body weight, respectively)—were able to restore and improve the morphology of pancreatic islets, as did glibenclamide at a 5 mg/kg dose.

### 2.7. UPLC-QTOF/ESI-MS Characterization of CILEx

**Figure 5:**
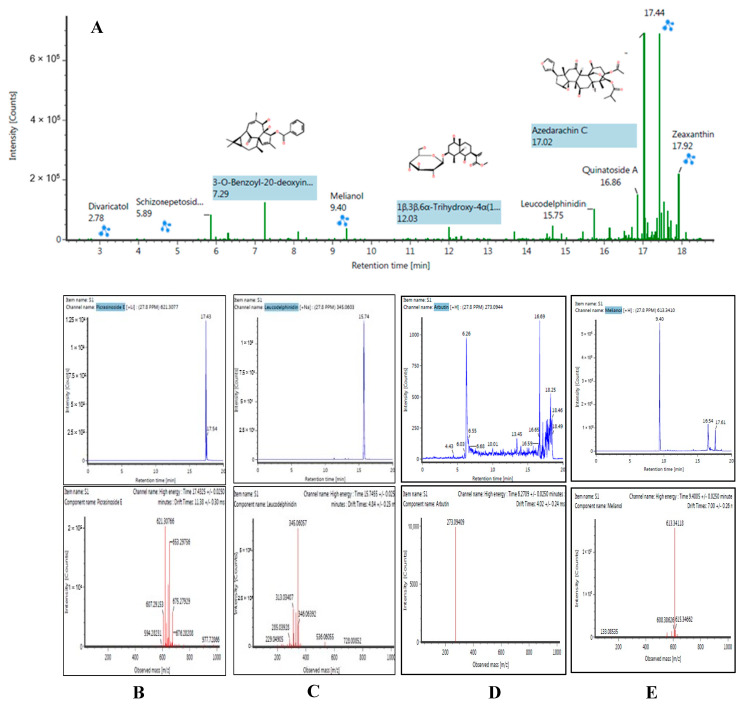
(**A**) Full chromatogram of CILEx in negative ion mode. LC chromatograms and mass spectra of CILEx for high-intensity compounds identified and vertically arranged as (**B**) picrasinoside E, (**C**) schizonepetoside E, (**D**) melianol, and (**E**) arbutin.

Ultra-performance liquid chromatography coupled with time-of-flight mass spectrometry (UPLC-QTOF/ESI-MS) is one of the most promising tools for the analysis of the phytochemical constituents in extracts. It can effectively separate and analyze the compounds by giving the inclusive mass of different ions and accurate chemical formulae [25]. In the present study, flavonoids, flavonoid glycosides, saponins, and terpenoids were identified by using either positive mode ((+) ESI-MS) or negative mode ((−) ESI-MS). The presence of compounds was determined on the basis of the pattern of mass fragments, low mass error (±5 mDa), and ion response. Automatic elucidation of fragment ions by mass fragments eases the process of verification. The identified compounds were classified as a good match with ±5 mDa error, or a poor match with ±10 mDa error, by the UNIFY software. A list of identified flavonoids, flavonoid glycosides, saponins, and terpenoids is shown in detail in Table 2, while the full liquid chromatogram and the mass spectra of high-intensity compounds identified are presented in Figure 5A. Chromatograms for the most promising individual compounds are presented in Figure 5B–E.

**Figure 6:**
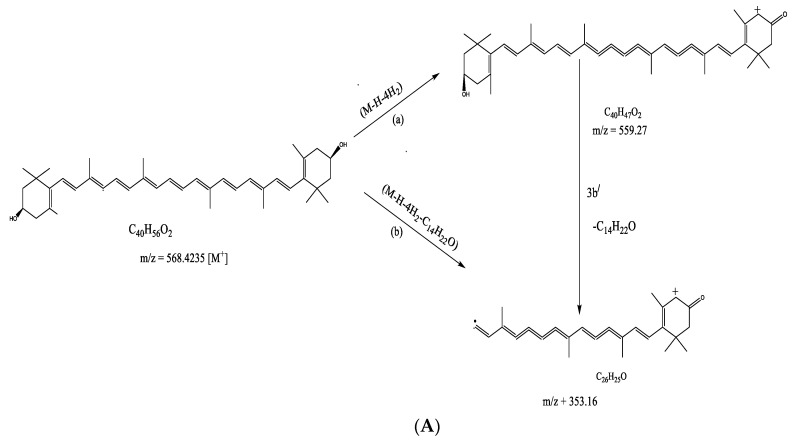

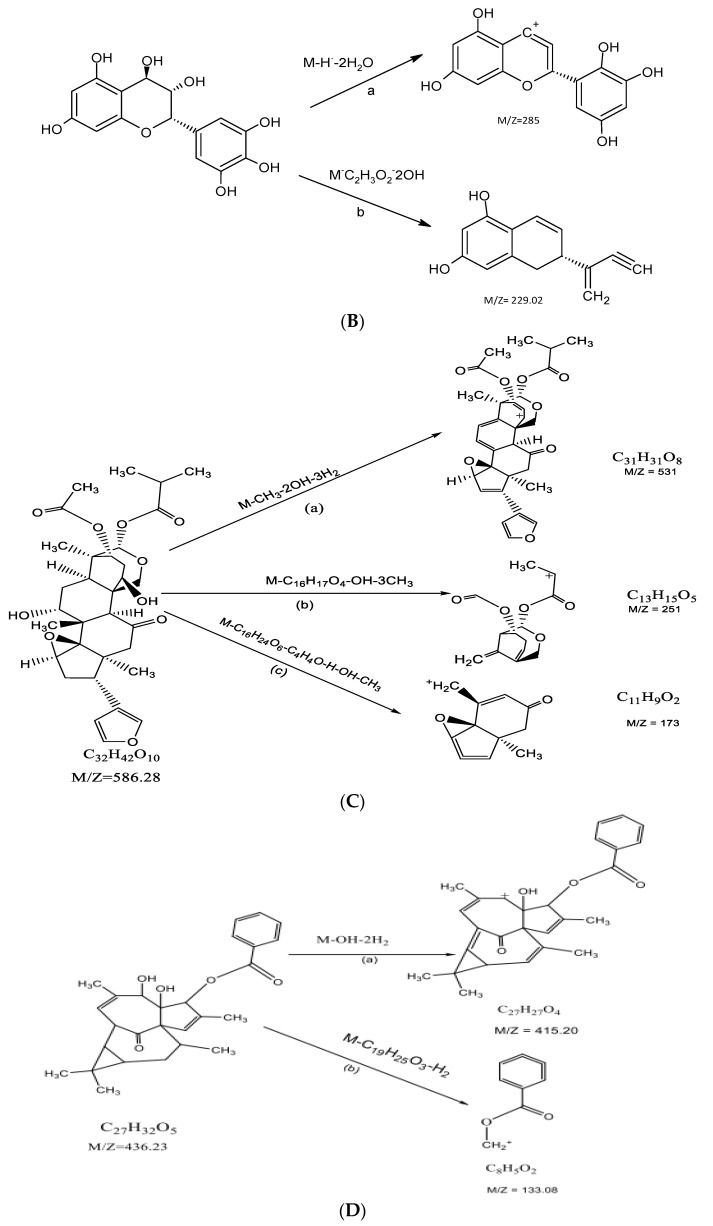

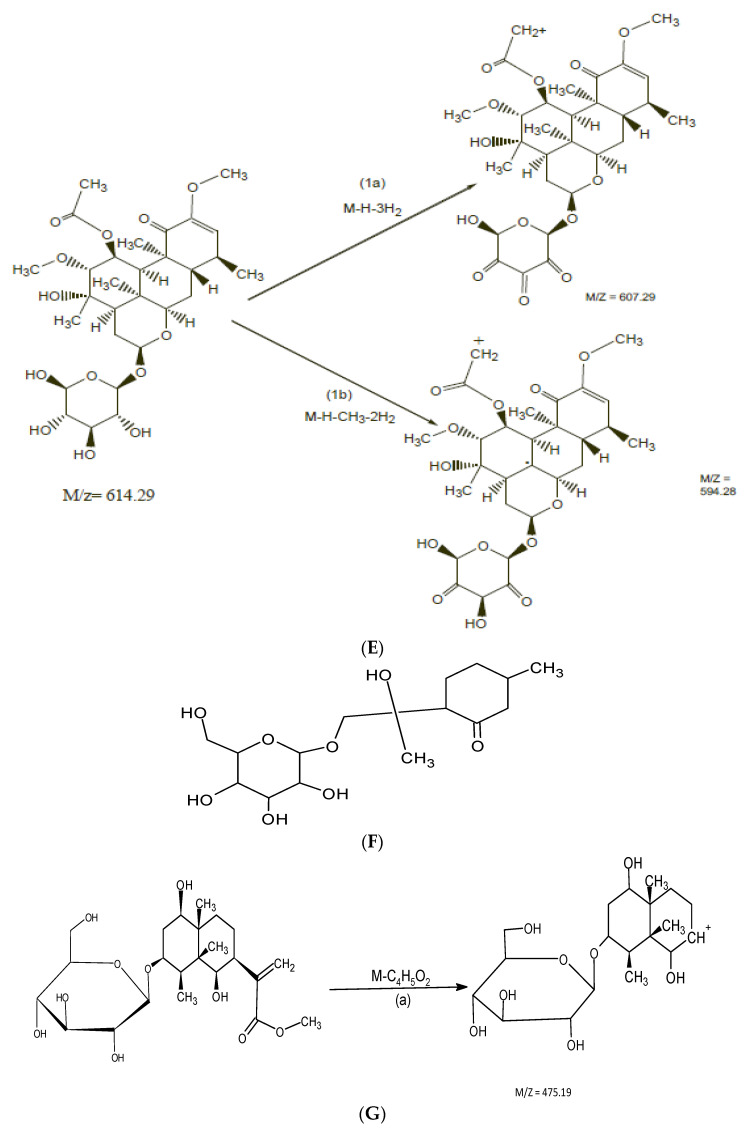

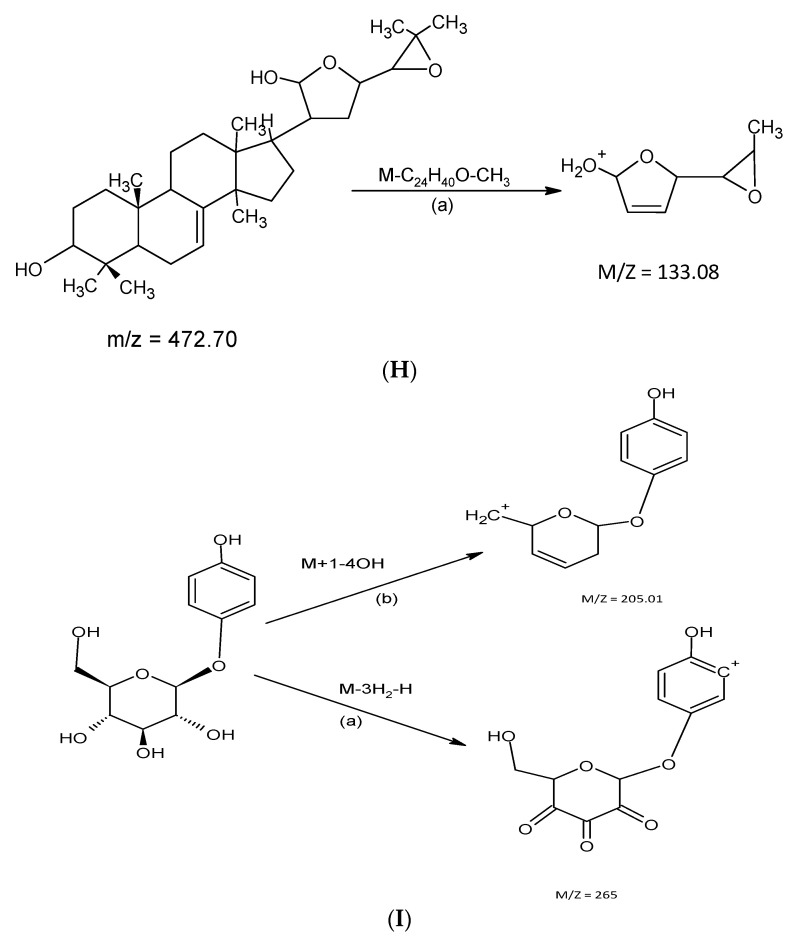
Mass fragmentation mechanisms of (**A**) zeaxanthin, (**B**) leucodelphinidin, (**C**) azedarachin C, (**D**) 3-*o*-benzyl-2-*o*-deoxyingenol, (**E**) picrasinoside E, (**F**) schizonepetoside E, (**G**) 1β,3β,6α-trihydroxy-4α(15)-dihydrocostic acid methyl ester-1-*o*-β-d glucopyranoside, (**H**) melianol, and (**I**) arbutin, as identified in the CILEx.

The mass spectrum of zeaxanthin displays a molecular ion (M+) peak at 568.4235, which undergoes a fragmentation to give a peak at *m*/*z* 559.27. corresponding to fragment ion [M–H–4H_2_] in Figure 6A (Path 1), which was further cleaved to make a daughter ion [M–H–4H_2_–C_14_H_22_O] at *m*/*z* 353.16 by removal of a fragment C_14_H_22_O (Path 2). The compound leucodelphinidin did not show any molecular ion peak corresponding to *m*/*z* 322.07. However, the molecular ion produced an ion [M–H–2H_2_O] in Figure 6B (Path 1) at *m*/*z* 285.03 upon the expulsion of one H radical and successive removal of two H_2_O molecules. Furthermore, the molecular ion resulted in a daughter ion [M–C_2_H_2_O_2_–H-2OH] (Path 2) at *m*/*z* 229.02 upon the removal of a C_2_H_2_O_2_ fragment by homolytic fission, followed by successive expulsion of one H radical and two OH radicals. Azedarachin C did not give any peak at *m*/*z* corresponding to molecular ion M+. However, it produced a fragment [M–CH_3_–2OH–3H_2_] (Figure 6C) (Path 1) at *m*/*z* 531 via the removal of one CH_3_ radical and two OH- (hydroxyl) radicals, followed by the expulsion of three H_2_ molecules. The other fragment [M–C_16_H_17_O_4_–OH–3CH_3_∙] in Figure 6C (Path 2) resulted from the cleavage to remove a large fragment C_16_H_17_O_4_, and the successive removal of one OH radical and three CH_3_ radicals at *m*/*z* 251. On the other hand, the molecular ion underwent cleavage to afford a daughter ion [M–C_16_H_24_O_6_–C_4_H_4_O–H–OH–CH_3_] in Figure 6C (path 3) at *m*/*z* 173 by removing two large fragments C_16_H_24_O_6_ and C_4_H_4_O, followed by the expulsion of one H radical, one OH radical, and one CH_3_ radical. The compound 3-*o*-benzoyl-2-*o*-deoxyingenol shows (M + 1) and (M + 2) peaks at *m*/*z* 437.23 and 438.23, respectively. A fragment [M–OH–2H_2_] 6D (Path 1) at *m*/*z* 415.20 was observed due to the successive removal of one OH radical and two molecules of H_2_. Further double cleavage (Path 2) of the compound (Figure 6D) produces a fragment [M–C_19_H_25_O_3_–H_2_] at *m*/*z* 133.08 via the removal of a large C_19_H_25_O_3_ fragment, followed by the expulsion of one H_2_ molecule. Picrasinoside E in its mass spectrum did not reveal any peak at the *m*/*z* value corresponding to its molecular ion M+. However, one fragment ion [M–H–3H_2_] at *m*/*z* 607.29 in Figure 6E (Path 1) may be the result of the successive expulsion of three H_2_ molecules and one H radical. The ion [M–H–2H_2_–CH_3_]^+^ in Figure 6E (Path 2) at *m*/*z* 594.28 can be attributed to the fragment resulting from the removal of one H radical, two H_2_ molecules, and a CH_3_ radical. In Figure 6F, schizonepetoside E afforded (M + 1) and (M + 2) peaks corresponding to *m*/*z* 349.18 and 350.18, respectively, instead of a molecular ion peak at *m*/*z* 348.18. It is surprising to note that this compound did not undergo any fragmentation. The compound 1β,3β,6α-trihydroxy-4α(15)-dihydrocostic acid methyl ester-1-*o*-β-d glucopyranoside revealed (M + 1) and (M + 2) peaks at *m*/*z* 461.23 and 462.23, respectively. Furthermore, the molecular ion cleaved to create a fragment ion [M–C_4_H_5_O_2_] at *m*/*z* 375.19 in Figure 6G, via the removal of a C_4_H_5_O_2_ fragment. Figure 6H shows the fragmentation pattern of melianol, which did not reveal any molecular ion peak at *m*/*z* 472.70, even when a fragment for this compound was identified. Importantly, the LCMS produced molecular weight does not befit with the molecular formula which makes the current fragmentation proposal ambiguous for future research. Finally, arbutin displays an (M + 1) peak at *m*/*z* 273.09, which upon successive removal of 4OH radicals can result in a daughter ion [M + 1 − 4OH] (Figure 6I (path 1)) at *m*/*z* 205.01. On the other hand, upon the removal of one H radical and three H_2_ molecules, the molecular ion produced an ion [M–H−3H_2_] (Figure 6I (Path 2)) at *m*/*z* 265.

### 2.8. Molecular Docking

**Figure 7:**
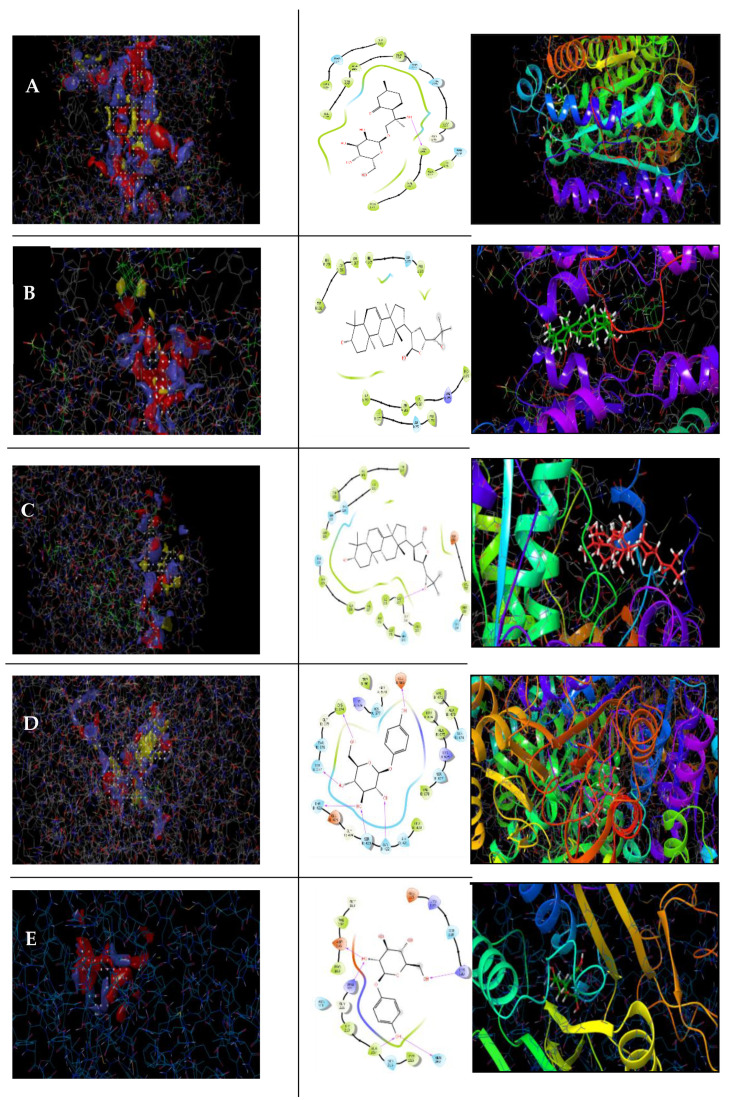
Based on site maps (**left**), interactions of five receptor proteins with seven ligands (**right**). Highest docking scorers are shown: (**A**) schizonepetoside showed the highest docking score (−8.145) with IXU9; (**B**) melianol (highest docking score, −8.475) with receptor IXU7; (**C**) melianol (highest docking score −8.995) with receptor 2BEL; (**D**) arbutin (highest docking score, −7.492) with receptor GR4F, and (**E**) 3-*o*-benzoyl-20-deoxyingenol (highest docking score, −6.123) with receptor 3A5J. In each case, gliclazide and metformin were used as reference antidiabetic drugs (Supplementary Materials).

The phytochemicals picrasinoside E, azedarachin C, arbutin, 3-*o*-benzoyl-20-deoxyingenol, leucodelphinidin, melianol, schizonepetoside E, zeaxanthin, reference drugs metformin and gliclazide, and 1XU9, 1XU7, 6R4F, and 3A5J were used as the receptors for molecular interaction. The predicted active sites for all of the target proteins are shown in Table 3. All of the ligands were bound to the receptor and produced scores, except for zeaxanthin. The proteins yielded multiple binding sites, and we used the sites with the best scores for each protein. Glide-docking resulted in scores for different parameters, including docking score, glide emodel, and glide energy, the three of which were used to evaluate the docking study presented in Table 4. The two-dimensional binding of the highest affinity compounds based on the site map interactions is displayed in Figure 7.

### 2.9. Analysis of Interactions between Active Ingredients and Target Proteins

**Figure 8:**
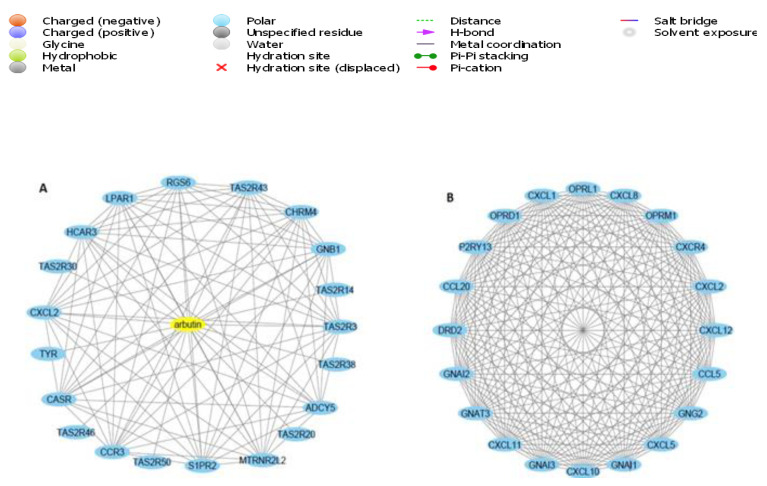
(**A**) CILEx arbutin–target interaction network—the yellow node represents the arbutin and the blue node represents the top 20 proteins—and (**B**) protein–protein interaction (PPI) network of top 20 ranked target proteins.

According to the previous analysis in this study, the active ingredients of CILEx show good pharmacological lipolytic and antidiabetic effects in a synergistic way. Arbutin, in this study, was found to interact significantly (PPI enrichment *p*-value <1.0 × 10^−16^) with 205 target proteins (Supplementary Table S1). Target proteins with the highest confidence scores for arbutin are displayed in Figure 8A. Cytoscape 3.6.1 was used to analyze the interaction between arbutin and the top 20 target proteins. Based on the arbutin–target protein relationships, it is now clear that arbutin acts on the target proteins. Recently, it was stated that arbutin alleviates diabetic symptoms by attenuating oxidative stress in mice through inhibiting the increasing blood glucose [26]. These results indicate that CILEx performs substantial biological and physiological activities via arbutin’s multitarget interactions.

### 2.10. Construction and Analysis of Target Proteins’ PPI Network

PPI networks play substantial roles in molecular processes, and abnormal PPI is the basis of many pathological conditions [27]. Using the STRING42 database and Network Analyst software [28], all target proteins (205) were mapped into the PPI network. Interestingly, we found that 203 target proteins are involved in the PPI network, with 17,310 edges, and an average node degree of 169, while the PPI enrichment *p*-value was less than 1.0 × 10^−16^. In this PPI network, the larger the node degree, the stronger the relationship between the proteins corresponding to the node in this network, which indicates that the target proteins play a key role in the whole interaction network, highlighting their importance. Only the HELZ and MTRNR2L proteins were not included in the PPI network. We delineated two subnetworks in the PPI network: subnetwork 1 included 200 target proteins (listed with degree of interaction in Supplementary Table S2), while subnetwork 2 included only 3 target proteins (ERCC1, ERCC4, and CHEK2). The 20 top ranked target proteins, along with their greatest degree of interactions with other proteins, are illustrated in Figure 8B. Cytoscape 3.6.1 was used to analyze the interaction among the top 20 target proteins. GNAI1, the top hub target, is one of the crucial genes for type 2 diabetes [29]. Figure 8B shows that most of the immunological target proteins—including CCL4, CXCR4, CXCL12, CXCL8, CXCL10, CXCL1, CXCL11, CXCL5, CCL20, and CXCL2—are centrally located in the PPI networks, with top degrees of interaction, indicating that this PPI network is associated with immunological activities. It has been stated that human chemokines are associated with or implicated in the pathogenesis of type 1 diabetes [30].

### 2.11. Gene Ontology (GO) Analysis of Interacted Target Proteins

**Figure 9:**
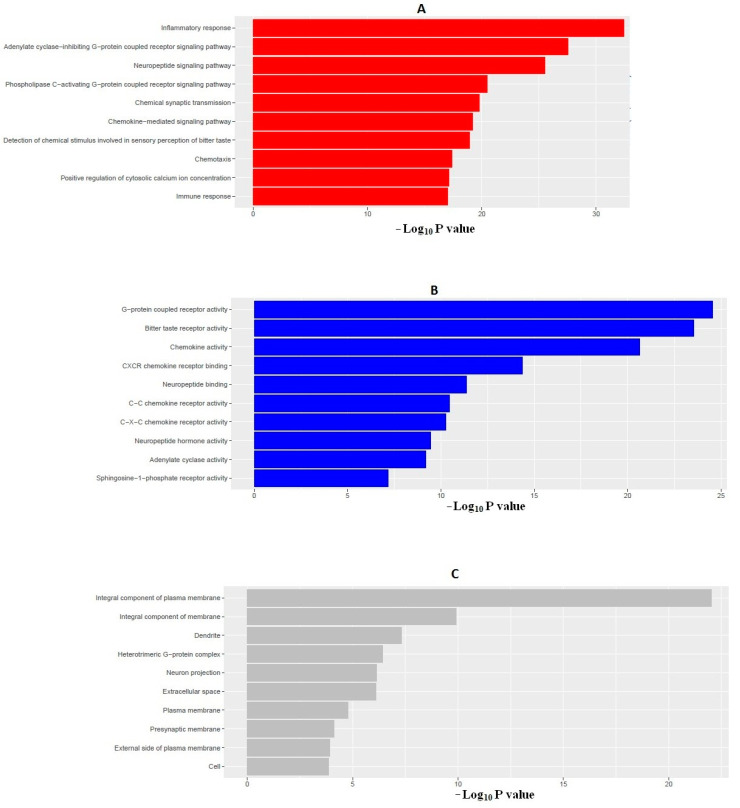
Gene Ontology (GO) enrichment analysis of the interacted target proteins: (**A**) top 10 biological processes (red); (**B**) top 10 molecular functions (blue); and (**C**) top 10 cellular components (gray).

GO enrichment analysis of interacted target proteins (total 203) that act with arbutin was performed using DAVID (https://david.ncifcrf.gov/, accessed on 3 August 2020). The top 10 significantly enriched terms in the biological process (BP), molecular function (MF), and cellular component (CC) categories were selected, according to Benjamini–Hochberg corrected *p*-values < 0.05. A total of 61 significant BPs are listed in Supplementary Table S3, and the top 10 BPs are represented in Figure 9A. In BP analysis, the target proteins are mainly involved in inflammatory response, immune response, and some other metabolic processes. We also found 31 significant molecular functions (Supplementary Table S4), the top 10 of which are illustrated in Figure 9B, including G-protein-coupled receptor activity and many chemokine-mediated immune responses. In addition, we also identified 10 significant CCs, as shown in Figure 9C and Supplementary Table S5, which mainly included integral components of the plasma membrane, heterotrimeric G-protein complex, extracellular space, exterior of the plasma membrane, and cell. Inflammatory response—the most significant biological process—is documented to be associated with diabetes [31]. G-protein-coupled receptor signaling pathways are related with the crosstalk with insulin signaling [32], while chemokines have been found to be associated with or implicated in the pathogenesis of type 1 diabetes [30]. Type 2 diabetes has broad impact on immune responses [33]. The GO analysis indicates that the target proteins may bind with the plasma membranes of cells to mediate the process of immunological activities, so as to exert the anti-inflammatory and antidiabetic potential of arbutin in CCs.

### 2.12. Target Proteins Set Enrichment Analysis of KEGG Pathways

**Figure 10:**
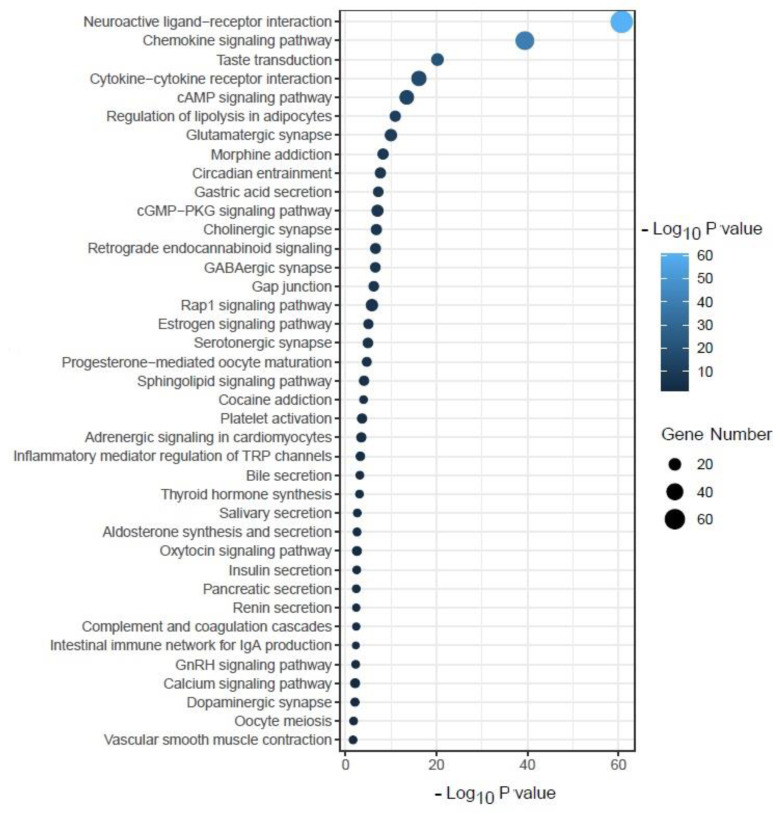
Some of the enriched KEGG pathways significantly associated with target proteins.

To further elucidate the relationship between the target proteins and the pathways, we identified 48 KEGG pathways that were significantly associated with the target proteins (Figure 10 and Supplementary Table S6). These pathways were mainly involved in immune regulation (chemokine signaling pathway, cytokine–cytokine receptor interaction, platelet activation, inflammatory mediator regulation of TRP channels, complement and coagulation cascades, and intestinal immune network for IgA production), secretion (gastric acid secretion, bile secretion, salivary secretion, aldosterone synthesis and secretion, insulin secretion, pancreatic secretion, and renin secretion), neurological regulation (neuroactive ligand–receptor interaction, taste transduction, glutamatergic synapse, morphine addiction, circadian entrainment, cholinergic synapse, GABAergic synapse, serotonergic synapse, cocaine addiction, and dopaminergic synapse), metabolism (regulation of lipolysis in adipocytes, thyroid hormone synthesis), cellular development (gap junction, progesterone-mediated oocyte maturation, oocyte meiosis, and vascular smooth muscle contraction), and cellular signaling (cAMP and cGMP-PKG signaling pathways, retrograde endocannabinoid signaling, Rap1 signaling pathway, estrogen signaling pathway, sphingolipid signaling pathway, adrenergic signaling in cardiomyocytes, oxytocin signaling pathway, GnRH signaling pathway, and calcium signaling pathway).

It was found that the neuroactive ligand–receptor interaction was predicted to be a major modulated pathway in an antidiabetic study [34]. Chemokines have been associated with or implicated in the pathogenesis of type 1 diabetes [30], while cytokines are crucial immunotherapeutic targets in diabetes [35].

### 2.13. Target Proteins Involved in Regulating the Diabetes-Associated Pathways

**Figure 11:**
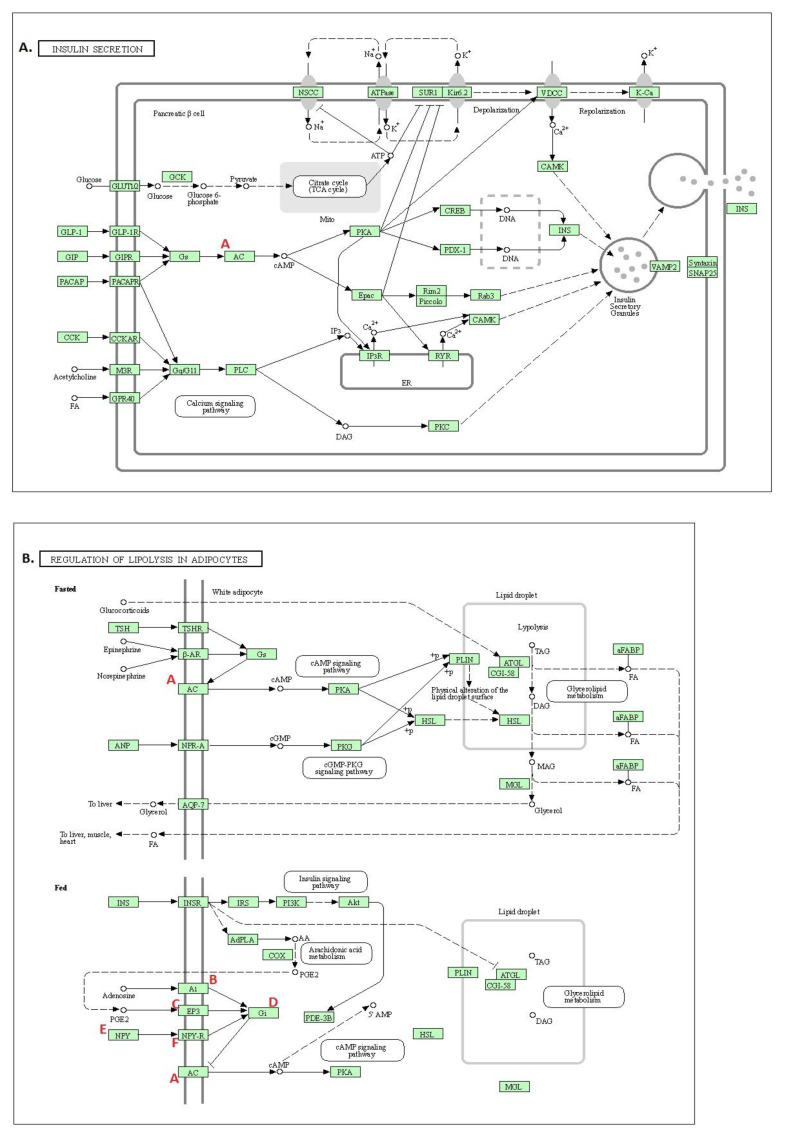
Involvement of target proteins in (**A**) insulin secretion and (**B**) regulation of lipolysis in adipocytes. A = ADCY1, ADCY3, ADCY4, ADCY5, ADCY6, ADCY7, ADCY8, and ADCY9; B = ADORA1; C = PTGER3; D = GNAI1, GNAI2, and GNAI3; E = NPY; F = NPY1R.

We found various pathways associated with diabetes (Figure 10 and Supplementary Table S6). For example, target proteins ADCY1, ADCY3, ADCY4, ADCY5, ADCY6, ADCY7, ADCY8, and ADCY9 are associated with insulin secretion (Figure 11A). Similarly, these target proteins are also associated with pancreatic secretion, bile secretion, and gastric acid secretion (Supplementary Table S6). In addition, ADCY3, ADCY4, ADCY1, PTGER3, GNAI3, GNAI2, ADCY7, GNAI1, ADCY8, ADCY5, ADCY6, NPY1R, ADORA1, NPY, and ADCY9 are linked with the regulation of lipolysis in adipocytes (Figure 11B). Gastric acid secretion is correlated with diabetic pathophysiology [36]. Fat cell lipolysis (i.e., fat cell triacylglycerol breakdown into fatty acids and glycerol in the absence of stimulatory factors) is elevated during obesity, and is correlated with insulin resistance [37].

## 3. Discussion

An effective in vivo model for antidiabetic testing ensures reliable results to evaluate the antidiabetic activity of plant extracts and compounds. Diabetes in rats was caused by alloxan, which is a popular and long-established agent for the induction of type 1 diabetes mellitus. The evidence from experiments and clinical studies reveals that the reactive oxygen species (ROS) level is higher in both type 1 and type 2 diabetes, playing prominent role in the development and progression of diabetic complications. The administration of alloxan causes sudden and drastic insulin secretion in the presence or absence of glucose. As a consequence, total suppression of beta cells occurs by inhibiting the glucose-sensing glucokinase enzyme. Thus, beta cells fail to recognize the glucose levels in the blood. Alloxan also produces ROS and superoxide free radicals, leading to the rapid damage of the islets of beta cells [38]. Elevation of ROS occurs via glucose autoxidation, and glucose–protein reaction increases glycation [39]. Anabolic enzymatic cofactors such as nitric oxide synthase (NOS), nicotinamide adenine diphosphate (NADP) oxidase, and xanthine oxidase are the sources of reactive species in diabetics, leading to various associated complications. Hence, the use of antioxidants would be one of the effective measures to reduce oxidative damage in diabetes.

Studying with a plant extract, plant-based product, or any formulation thereof requires the evaluation of their safety profiles; this is particularly important for unknown plant extracts or compounds. The acute oral toxicity test in animal models helps select the safe dose that could be extrapolated in human clinical trials. However, different pharmacokinetic behavior could still be observed in humans than in animals. It has been reported that different pathophysiological disorders related to the gastrointestinal tract and blood in animals have similarities with those of humans [40]. Thus, the acute oral toxicity study helps measure various toxic effects of the extract when given in a single dose, which is also useful for the researchers to adjust the doses in an experiment. Our observation shows that CILEx leaf ethanol extract has no toxic effects when given at a higher dose (1000 mg/kg body weight).

A significantly reduced fasting blood sugar of the treatment groups in this study was displayed throughout the study period due to the administration of CILEx (250 and 500 mg/kg body wt.), which potentially improved blood sugar levels in alloxan-induced diabetic rats. The increased oxidative stress combined with increased blood glucose levels and free fatty acids badly affect insulin secretion and function [41]. The extract might have shown a beneficial effect on the islet of beta cells, as well as antioxidant activity through the high total phenolic and flavonoid contents of CILEx. Additionally, plant polyphenols exhibit antioxidant properties by donating hydrogen from their hydroxyl groups [42] and, thus, participate in the antioxidant activity. Previous reports revealed a positive correlation between the phenolic contents and antioxidant activity of *C. sericeum* and *C. acutum* [43]. Other studies have also reported the antioxidant activity of certain *Combretum* species [44]. Therefore, the use of antioxidants in the treatment of diabetes-associated complications has been an effective strategy of choice [39]. Some natural antioxidants—such as vitamin C, vitamin E, and α-lipoic acid treatments—were found to reduce the oxidative stress in animals, as well as in humans [39].

In diabetes mellitus, an abnormal lipid profile is a common manifestation. Literature suggests that hyperlipidemic conditions are among the most common consequences of alloxan induction in experimental rats [45]. In our study, elevation of total cholesterol levels was observed in diabetic rats, which was probably due to elevated free fatty acid levels having harmful effects in the body through free radical accumulation and stimulation of protein kinase C [46]. Hyperlipidemia causes decreased glucose transport to the cells, making lipids available in the form of LDL fat, which deposits in the blood vessels and is transported to the liver by HDL for elimination. Thus, the increased HDL and decreased LDL levels are expected to be beneficial for the therapeutic application of CILEx, which was achieved in this study.

Any abnormality of the architecture of the pancreatic tissue may alter the secretion, sensitivity, and function of insulin from islets of beta cells. Cellular atrophy and degeneration of pancreatic beta cells are marked as damage to the pancreas [47]. In the diabetic control group of this study, the number of beta cells was reduced and shrinkage was seen, while the normal control group retained regular cellular integrity. In the treatment groups, the beta cells were well recovered compared to in the normal rats.

The phytochemical composition of CILEx was characterized using the UPLC-QTOF/ESI-MS technique. This helps in the profiling and subsequent standardization of phytochemicals in the extract. UPLC-QTOF/ESI-MS analysis shows that the ethanol leaf extract contains a complex mixture of flavonoid, glycosides, saponins, and terpenoids of different classes. Among the 71 identified compounds, 6 were previously reported for their hypoglycemic and lipid-lowering effects. It has been reported that arbutin significantly inhibits α-amylase and α-glucosidase activity in vitro [28]. Arbutin-rich *Pyrus boissieriana* Buhse leaves also reduced glucose and lipid levels in blood, with an increased antioxidant state in alloxan-induced hyperglycemic rats [48]. Geetha et al. revealed that leucodelphinidin was found in the bark of *Ficus benghalensis*, and exerted hypoglycemic effects on either normal or diabetes-induced rats [49]. Saponins are known for their ability to lower plasma cholesterol levels and the risk of many chronic diseases in humans [50]. Ginsenoside Rh2—a glycosylated triterpene—when administered in fructose-rich chow-fed rats, causes plasma glucose to fall with enhanced insulin sensitivity and secretion [51]. Early literature also reveals that loganin, lycopene, and zeaxanthin have significant hypoglycemic effects in diabetic rats, and decrease fasting blood glucose levels in diabetes mellitus mice. The supplementation of lycopene significantly reduces diabetic plasma glucose levels [52].

Arbutin (ARB) has been associated with protecting HK-2 cells against high-glucose-induced apoptosis and autophagy in diabetic nephropathy (DN) through regulating the miR-27a/JNK/mTOR axis [53]. In CC analysis, we found that target proteins are associated with membranes, while in MF analysis, we found that many of the target proteins were linked with G-protein-coupled receptors. It has been stated that islet G-protein-coupled receptors are potential therapeutic targets for diabetes [54]. Thus, ARB targeted proteins may be associated with antidiabetic activities through membrane-receptor-mediated cellular signaling. Pathway analysis further reveals that ARB is associated with insulin secretion and pancreatic secretion. Altogether, our analyses indicate that CILEx is a potential antidiabetic agent [55]. These results indicate that CILEx exerts its antidiabetic and lipid-lowering activities through potentially regulating insulin secretion, pancreatic secretion, and lipolysis regulation in adipocytes.

## 4. Materials and Methods

### 4.1. Chemicals and Reagents

Analytical grade chemicals and reagents were used in this research, except where specified otherwise. Ethanol, alloxan, gallic acid, potassium acetate and quercetin were obtained from Sigma-Aldrich Chemicals, CA, USA. Glibenclamide (Chadwell Heath Essex, England), DPPH (1,1-diphenyl-2-picrylhydrazyl), ascorbic acid, Folin–Ciocalteu reagent, and sodium carbonate were purchased from Merck, India, while aluminum chloride was procured from Fine Chemicals, Delhi, India.

### 4.2. Collection and Identification of Plant Material

The fresh leaves of *Combretum indicum* (CILEx) were collected in April 2018 from Tangail District. The plant was identified by Professor Dr. Sheikh Bokhtear Uddin, a taxonomist at the Department of Botany, University of Chittagong, Bangladesh. A voucher specimen (accession No. 47044) has been deposited to the Bangladesh National Herbarium, Mirpur, Dhaka for future reference.

### 4.3. Preparation of Crude Extract

The fresh leaves of CILEx were washed with distilled water and shade-dried for 7 days at room temperature. The dried leaves were ground to powder (500 g) using a mechanical grinder (Miyako, Model No: DL-718 Jiaxing China) and stored in an airtight container. Then, 500 g of dried powder was soaked in 2500 mL of 96% ethanol at room temperature (25 ± 1 °C), with occasional stirring. After 14 days, the extract was filtered and concentrated in vacuum using a rotary evaporator (Barloworld, Berkshire, UK). The concentrated extract was then allowed to air dry for complete evaporation of the solvent. Finally, a blackish-green semisolid extract was preserved at 4 °C until further use.

### 4.4. Experimental Animals and Their Maintenance

Long–Evans rats (26 rats; age: 5–6 weeks; average body weight: 92 ± 9 g) of both sexes were obtained from the International Centre for Diarrheal Disease Research, Bangladesh (ICDDR, B), Mohakhali, Dhaka, Bangladesh. During the experimental period, the rats were kept in a well-ventilated animal house at room temperature and were supplied with a standard commercial rat pellet diet from ICDDR, B, and fresh drinking water. The animals were housed in plastic cages, and soft wood shavings were used as bedding. Animals were maintained under standard environmental conditions (temperature: 25 ± 1 °C; relative humidity: 55–65%; and a 12 h/12 h day/night cycle) in a properly ventilated room. Animals were handled and maintained according to the local animal ethical guidelines approved by the institutional animal ethics committee of Southeast University, Dhaka, Bangladesh (Approval No.: SEU/Pharm/CECR/102/2019).

### 4.5. Determination of Total Phenolic and Flavonoid Contents

A slightly modified Folin–Ciocalteu method was used to determine the total phenolic content (TPC) [56]. Briefly, a standard gallic acid (6.25–200 μg/mL) calibration curve was prepared, and leaf extract was prepared at a concentration of 200 μg/mL. Next, 1 mL of the extract solution or standard gallic acid solution was taken in a screw cap tube, and 5 mL of Folin–Ciocalteu reagent (previously prepared as 10% *v*/*v* dilution in distilled water) was added. Then, 4 mL of anhydrous sodium carbonate (7.5%) was added and incubated for 30 min at 40 °C. A typical blank solution contained the vehicle solvent. Absorbance was taken at 765 nm with a UV–Vis spectrophotometer (Shimadzu, Kyoto, Japan). The total phenolic content was calculated as gallic acid equivalent (GAE) by the following equation:

C = (c × V)/m

where C = TPC (mg/g plant extract in GAE), c = the concentration of the sample obtained from the calibration curve (mg/mL), V = the volume of the sample, and m = the sample weight (g).

The total flavonoid content (TFC) of CILEx was determined according to the method of Rahman et al. [57].

### 4.6. DPPH Radical Scavenging Assay

The free radical scavenging effect of CILEx was evaluated with the stable scavenger DPPH described by Rahman et al. [58]. Briefly, 100 μL of CILEx and standard (ascorbic acid) solution in different concentrations was taken, and 3 mL of DPPH solution (0.004%) was mixed separately. These solutions were kept in the dark for 30 min to read absorbance at 517 nm using a UV–Vis spectrophotometer. Lower absorbance of the reaction mixture indicated higher free radical scavenging activity. Percentage inhibition was determined by the following formula:

Percentage of scavenging activity (%) = [(A − B)/A] × 100, where A is the absorbance of the control (DPPH solution without the sample), and B is the absorbance of the DPPH solution in the presence of the sample (extract/ascorbic acid). Then, % scavenging was plotted against concentration, and IC_50_ was calculated.

### 4.7. Acute Oral Toxicity Test

Acute oral toxicity testing of CILEx was performed on Long–Evans rats, according to OECD-423 guidelines (acute toxic class method), with slight modifications. The animals were overnight fasted, providing only water. Two groups of three rats each were used for this study. Group I received a single oral dose of CILEx (500 mg/kg body weight), and Group II received a single oral dose of CILEx (1000 mg/kg body weight). After the oral administration of CILEx, animals were observed individually at least once in the first 30 min, and periodically over the first 24 h, with special attention given during the first 4 h, for 10 consecutive days. All observations were systematically recorded for each animal. The animals were observed for gross behavioral, neurological, and autonomic effects. Additional conditions such as tremors, convulsions, salivation, diarrhea, lethargy, sleep, coma, and lethality were also observed. The effective therapeutic dose was calculated as one-tenth of the median lethal dose using the arithmetic method of Karber G in association with the Hodge–Sterner scale (LD50 > 2.0 g/kg) (58).

### 4.8. Induction of Diabetes and Experimental Design

Sixteen Long–Evans rats (average body weight 92 ± 9 g) of both sexes were used for the induction of diabetes. Diabetes was induced in overnight-fasted rats by a single intraperitoneal (IP) injection of alloxan monohydrate (150 mg/kg). Two days after alloxan injection, fasting blood glucose levels of all of the animals were recorded from tail vein blood using a portable glucometer (Accu-Chek, Japan), and rats with plasma glucose levels of >7.5 mmol/L were confirmed for the study. Treatment with CILEx was started after 48 h of alloxan injection. The plant sample CILEx, standard glibenclamide, and saline were administered with the help of feeding cannulas. Fasting blood glucose estimation was carried out on days 3, 5, and 7 of the study. The animals were grouped as follows:

Normal control (I): Normal rats received saline water only.
Diabetic control (II): Non-treated diabetic rats (alloxan treated; 150 mg/kg; IP).
Positive control (III): Alloxan-treated diabetic rats (150 mg/kg; IP) + glibenclamide (5 mg/kg; PO)
Treatment group (IV): Alloxan (150 mg/kg; IP) + CILEx (250 mg/kg; PO)
Treatment group (V): Alloxan (150 mg/kg; IP) + CILEx (500 mg/kg; PO)

### 4.9. Collection of Blood and Serum Analysis

After 7 days of treatment, animals were fasted for 12 h and their blood glucose levels were measured. The animals were then anaesthetized using diethyl ether and euthanized by decapitation. Blood was collected in a dry test tube from cardiac vessels using a disposable syringe via the heart puncture method [59], centrifuged (Hitachi, Japan) at 112 g for 15 min, and then the plasma samples were stored at 4 °C until biochemical estimations. Total cholesterol (TC), triglyceride (TG), high-density lipoprotein (HDL), and low-density lipoprotein (LDL) were measured using wet reagent diagnostic kits according to the manufacturer’s protocol, using a biochemistry analyzer (BAS 100TS, Spectronics Corporation, LA, U.S.A) [60,61,62,63].

### 4.10. Histopathological Studies

After euthanizing the animals, pancreases of two animals from each group were excised and stored in 10% buffered formalin solution after washing with normal saline water. The pancreas was washed, dehydrated with alcohol, and cleared with xylene, and then paraffin blocks were made. Serial sections of 4–5 μm in thickness were cut using a microtome (semi-automated, Biobase BK-MT390S (BK-2488, Jinan, Shandong, China). Then, the sections were deparaffinized with xylene and hydrated in descending grades of alcohol. The slides were then transferred to hematoxylin for 10 min, followed by rinsing with water. These were examined and later stained with eosin, rinsed with water, dehydrated with ascending grades of alcohol, cleared with xylene, and mounted. Different parameters of pancreatic cellular condition were observed under a compound microscope, and the histopathological images were taken with the help of an Optica DP20 system (Italy).

### 4.11. UPLC-QTOF/MS Analysis

The phytochemical profiling of the CILEx was determined using UPLC-MS. UPLC-MS analysis was performed using Waters ACQUITY UPLC IClass/Xevo in line with a Waters Xevo G2 Q-TOF mass spectrometer (Milford, MA, USA). Extract samples were prepared by dissolving 100 mg of CILEx in 1 mL of methanol. Separation was conducted on a Zorbax Eclipse plus Acquity UPLC BEH C18 (1.7 μm particle size) 2.1 mm × 50 mm. The UPLC was interfaced with a Q-TOF mass spectrometer integrated with positive and negative electrospray ionization (ESI) sources. Full-scan mode from *m*/*z* 50 to 1000 was performed with a source temperature of 120 °C. Solvent A was water with 0.1% formic acid, while solvent B was acetonitrile with 0.1% formic acid. A gradient elution was performed, starting with 99% solvent A and 1% solvent B for the first 15 min, and then 65% solvent A and 35% solvent B for 1 min, followed by a gradual increase in solvent A to 100% over 2 min, and finally a slow increase in solvent B to 99% and solvent A 1% over 2 min. Highly purified nitrogen (N_2_) and ultra-high-purity helium (He) were used as a nebulizing gas and collision gas, respectively. In terms of positive electrospray mode, the capillary voltage was set at 2.0 kV. Other instrument conditions implied were: source offset, 100 V; desolvation temperature at 550 °C; 50 L/h cone gas flow with temperature 120 °C; and desolvation gas flow, 800 L/h.

### 4.12. Computational Molecular Docking Analysis

#### 4.12.1. Preparation of Ligands

LigPrep (ver. 2018, New York, NY, USA) was used in this regard to prepare the ligands, and yielded 3D structures with accurate chiralities [64]. It generated possible states at a target pH of 7.0 ± 2.0 using Epik v4.6.12 [65], and also desalted and produced tautomers. Computationally, specified chiralities were retained and generated at a rate of 32 per ligand at most. Then, output was saved as Maestro on the device. All of the ligands were imported in SDF format from PubChem.

#### 4.12.2. Protein Preparation

Protein Preparation Wizard was used to modify the crystallographic structures of the proteins that were taken from the PDB (Protein Data Bank) [66]. The proteins 1XU9, 1XU7, 2BEL, 6R4F, 3A5J from the PDB were imported to Protein Preparation Wizard. Glide v8.1.12 was used in this regard, to optimize the structures from their raw state. The proteins were preprocessed by assigning bond orders using the CCD database, adding hydrogens, creating zero-order bonds to metals and disulfide bonds, deleting waters beyond 5.00 Å from the het groups, and generating het states using Epik at pH of 7.0 ± 2.0. The H-bond assignment was done by orienting water molecules, (Epik v4.6.12, Schrödinger, LLC, 2018-4, and PROPKA) at a specific pH of 7.0 by Schrödinger Release 2018-4 (SiteMap, Schrödinger, LLC, 2018-4), to determine the states of protonation, and to predict the pKa values of the residues [67,68]. Restrained minimization was done by converging heavy atoms to RMSD 0.30 Å.

#### 4.12.3. SiteMap: Active Site Prediction

The proteins 1XU9, 1XU7, and 6R4F had multiple binding sites, while 3A5J did not have any binding sites for ligand–protein interaction. To look for the possible binding sites, we used SiteMap from Schrödinger, 2018-4 [69], so that the ligands could bind to the receptor tightly [70]. The tool produced the maps based on hydrophobic and hydrophilic (donor, acceptor, and metal-binding portions) maps. For selection of binding sites, SiteScore and druggability score (Dscore)—including site size, volume, exposure, enclosure, contact, hydrophobic and hydrophilic character, balance (phobic/philic ratio), and donor/acceptor of hydrogen bond—were used to evaluate each active site [71].

#### 4.12.4. Receptor Grid Generation and Molecular Docking

The sites visualized by SiteMap were used as the entry, and Glide v8.1.12 (Schrödinger, LLC, 2018-4) was used to discover the suitable interaction between a ligand and a protein [72]. Van der Waals radii of receptor atoms with partial charge (absolute value) were scaled at a scaling factor of 1.0 and partial charge cutoff of 0.25 to soften the potential for the non-polar part. Site constraints, rotatable groups, and excluded volumes were set to default settings, as provided by Maestro 11.8.

In ligand–receptor interaction, Van der Waals radii were fixed at a scaling factor of 0.80, and partial charge cutoff was scaled at 0.15 for the non-polar parts of the ligands. SP (standard precision) was set for ligand screening and sampling. The energy window for ring sampling to generate conformers was 2.5 kcal/mol. Initial poses for docking were kept at 5000 poses per ligand, and the scoring window was 100–400 poses for energy optimization. Post-docking minimization was performed for 5 poses per ligand, the strain-correcting threshold was 4.00 Kcal/mol, and excess strain energy was scaled at 0.25. The parameters used were defaults, as provided by Maestro 11.8 [73,74,75].

#### 4.12.5. Bioactive Compound–Target Protein Network Construction

On the basis of network pharmacology-based prediction, STITCH 5 (http://stitch.embl.de/, ver. 5.0, accessed on 3 August 2020) was used to identify target proteins related to the bioactive phytochemicals that were identified in CILEx [43]. It calculated a score for each pair of protein–chemical interactions. Chemical names of bioactive compounds (picrasinoside E, azedarachin C, zeaxanthin, quinatoside A, 3-*o*-Benzoyl-20-deoxyingenol, leucodelphinidin, schizonepetoside E, 1β,3β,6α-Trihydroxy-4α(15)-dihydrocostic acid methyl ester-1-*o*-β-d-glucopyranoside, melianol, and arbutin) were put into STITCH 5 individually to match their potential targets, with the organism selected as “Homo sapiens” and the medium required interaction score being ≥0.4.We predicted 205 target proteins with medium confidence score for arbutin, which was confirmed. The compound targets with no relationship with the compound–protein interactions were not considered for further analysis. The obtained compound–protein interaction data of the top 20 target proteins were imported into Cytoscape 3.6.1 software to construct a compound–protein interaction network.

#### 4.12.6. Construction of Protein–Protein Interaction (PPI) Network of the Predicted Genes

We constructed a PPI network of the predicted genes by using the search tool for the retrieval of interacting genes (STRING) database (https://string-db.org/cgi/input.pl; STRING-DB v11.0, accessed on 3 August 2020) [76]. The rank of the target proteins based on degree of interactions in the PPI network was identified using the node explorer module of NetworkAnalyst software [77]. The obtained protein interaction data of the top 20 target proteins were imported into Cytoscape 3.6.1 software to construct a PPI network.

#### 4.12.7. Gene Ontology (GO) and Kyoto Encyclopedia of Genes and Genomes (KEGG) Pathway Enrichment Analyses of the Target Proteins

To identify the role of target proteins that interact with the active ingredients of CILEx in gene function and signaling pathways, the Database for Annotation, Visualization, and Integrated Discovery (DAVID, https://david.ncifcrf.gov/, accessed on 3 August 2020) v6.8 was employed [78]. The KEGG [79] pathways significantly associated with the predicted genes were identified. We analyzed the Gene Ontology (GO) function and KEGG pathway enrichment of proteins (203 target proteins) involved in the PPI network. The target proteins involved in the cellular components (CCs), molecular functions (MFs), biological processes (BPs), and the KEGG pathways were also described. An adjusted *p*-value < 0.05, calculated by the Benjamini–Hochberg method, was considered to be significant [80].

### 4.13. Statistical Analysis

The data on fasting blood sugar and biochemical estimations were expressed as mean ± standard deviation (SD), and statistical comparisons were performed by one-way analysis of variance (ANOVA), followed by Tukey’s post hoc test, using GraphPad Prism (version 6 for Windows, GraphPad Software, San Diego, CA, USA, www.graphpad.com (accessed on 3 August 2020)). *p*-values less than 0.05 were considered to be significant.

## 5. Conclusions

The current study results indicate that *C. indicum* leaves have potential benefits for the treatment of diabetes and its associated complications, by protecting the pancreases through the normalization of damaged beta islets and improvement of the lipid profile. However, more studies are recommended in order to understand the mechanism and to isolate the bioactive compounds.

## Figures and Tables

**Table 1:** Antioxidative status of CILEx.

Indices	Values (Unit)	Reference/Standard
Total phenolic content	155 ± 7.35 mg/g as GAE	
Total flavonoid content	164.33 ± 2.71 mg/g as QE	80.65 ± 2.8 µg/mL
Inhibition concentration (IC_50_)	165.6 ± 3.1 µg/mL	

Data presented as mean ± SEM.

**Table 2:** Tentatively identified compounds of CILEx.

SL No.	RT (min)	Molecular Formula	Observed Mass (*m*/*z*)/Neutral mass	[M − H]^−^ (*m*/*z*)	Main Fragments	Compound
A.	17.92	C_40_H_56_O_2_	568.4282	567	593.27, 585.43,55 9.27, 353.16, 635.28, 636.28,683.33, 834.24	Zeaxanthin
B.	15.75	C_15_H_14_O_8_	345.0603	344	345.06, 313.03, 285.03, 229.04 346.06, 536.06, 728.00	Leucodelphinidin
C.	17.02	C_32_H_42_O_10_	609.2711	608	609.27, 591.26,531.23, 251.14, 173.09, 625.26, 667.27, 854.58	Azedarachin C
D.	7.29	C_27_H_32_O_5_	437.2363	436	437.23, 415.20 133.08, 438.23 459.21	3-*o*-Benzoyl-2-*o*- deoxyingenol
E.	17.44	C_30_H_46_O_13_	621.29	620	621.30, 607.29, 594.28, 653.29 675.27, 676.28, 977.72	Picrasinoside E
F.	5.89	C_16_H_28_O_8_	349.1834	348	349.18, 350.18	Schizonepetoside E
G.	12.03	C_22_H_36_O_10_	461.2358	460	461.23, 375.19, 462.23, 553.30 863.48	1β,3β,6α-Trihydroxy-4α(15)-dihydrocostic acid methyl ester-1-*o*-β-d-glucopyranoside
H.	9.40	C_35_H_48_O_9_	613.3410	612	613.34, 608.38, 133.08, 615.34	Melianol
I.	6.27	C_12_H_16_O_7_	273.0944	272	205.01, 265.14, 273.09	Arbutin

Note: The compounds are arranged according to their fragmentation details in the text.

**Table 3:** Active site prediction of enlisted proteins using SiteMap. Only the top ranked scores are mentioned on the table for each protein.

PDB ID	SiteScore	Size	Dscore	Volume	Exposure	Enclosure	Contact	Phobic	Philic	Balance	Don/acc
**1XU9**	1.069	666	0.969	1496.166	0.481	0.801	1.079	0.264	1.384	0.191	0.714
**1XU7**	1.05	182	0.904	336.14	0.504	0.772	1.06	0.199	1.532	0.13	0.33
**2BEL**	1.093	122	1.117	471.282	0.556	0.812	0.904	0.606	0.951	0.637	0.674
**6R4F**	1.155	308	1.12	766.262	0.274	0.929	1.195	0.762	1.157	0.659	0.628
**3A5J**	0.807	53	0.635	214.032	0.662	0.707	0.859	0.129	1.453	0.089	0.443

**Table 4:** Docking scores of 8 PubChem-available compounds out of 10 identified compounds from LCMS data for CILEx.

Compounds	Docking Score	Glide Model	Glide Energy
**1XU9**			
Metformin	−2.947	−25.206	−18.318
Gliclazide	−6.115	−49.527	−37.472
Picrasinoside E	−2.343	−17.099	−20.358
Azedarachin C	−2.575	−32.511	−28.533
Arbutin	−6.172	−46.152	−33.944
3−*o*−Benzoyl−20−deoxyingenol	−6.008	−44.674	−23.01
Leucodelphinidin	−6.468	−53.684	−39.835
Melianol	−8.363	−49.99	−36.079
Schizonepetoside E	−8.145	−59.072	−47.635
**1XU7**			
Metformin	−4.163	−29.164	−23.565
Gliclazide	−4.913	−34.925	−26.387
Picrasinoside E	−4.196	−39.489	−36.295
Azedarachin C	−5.662	−31.181	−34.502
Arbutin	−5.163	−37.436	−29.202
3−*o*−Benzoyl−20−deoxyingenol	−6.049	−35.397	−31.4
Leucodelphinidin	−6.166	−46.923	−35.177
Melianol	−8.475	−66.188	−47.383
Schizonepetoside E	−6.641	−47.271	−35.517
**2BEL**			
Metformin	−2.99	−22.089	−17.491
Gliclazide	−5.885	−53.992	−39.404
Picrasinoside_E	−6.358	−57.254	−45.617
Azedarachin_C	−7.246	−53.762	−42.76
Arbutin	−5.957	−49.719	−37.458
3−*o*−Benzoyl−20−deoxyingenol	−5.948	−54.747	−41.134
Leucodelphinidin	−6.744	−64.075	−45.816
Melianol	−8.995	−77.557	−52.946
Schizonepetoside_E	−7.685	−60.926	−44.759
**6R4F**			
Metformin	−4.031	−29.186	−19.423
Arbutin	−7.492	−74.393	−57.298
Leucodelphinidin	−5.279	−32.25	−23.607
Schizonepetoside_E	−6.859	−64.027	−53.418
**3A5J**			
Metformin	−2.71	−18.439	−16.472
Gliclazide	−4.252	−50.298	−36.799
Picrasinoside E	−4.055	−50.347	−42.864
Azedarachin C	−2.39	−29.319	−30.194
Arbutin	−4.962	−45.683	−35.8
3−*o*−Benzoyl−20−deoxyingenol	−3.532	−48.425	−40.094
Leucodelphinidin	−6.123	−58.309	−43.265
Melianol	−4.453	−52.271	−42.912
Schizonepetoside E	−5.716	−50.39	−44.064

## Data Availability

All data are available upon request to the authors.

## Supplementary Materials

Figure S1: LC chromatogram and mass spectra of CILEx for high-intensity compounds identified and vertically arranged as (**A**) Picrasinoside E; (**B**) Azedarachin C; (**C**) Zeaxanthin; (**D**) Quinatoside A; (**E**) 3-*o*-Benzoyl-20- deoxyingenol; (**F**) Leucodelphinidin; (**G**) Schizonepetoside E; (**H**) 1β,3β,6α-Trihydroxy-4α(15)-dihydrocostic acid methyl ester-1-*o*-β-Dglucopyranoside; (**I**) Melianol; (**J**) Arbutin., Figure S2: list of chemical structures of the ligand molecules available in PubChem., Figure S3: Interactions based on **A**. site map of seven ligands **D**. Picrasonide E; **E.** Azedarachin C; **F.** 3-*o*-Benzoyl-20-deoxyingenol; **G.** Leucodelphinidin; H. Schizonepetoside E; **I.** Melianol; and **J.** Arbutin with the receptor **IXU9**. Schizonepetosie showed the highest docking score (−8.145) compared with the reference antidiabetic drugs B. Gliclazide and C. Metformin., Figure S4: Interactions based on **A**. site map of seven ligands **D**. Picrasonide_E; **E.** Azedarachin C; **F.** 3-*o*-Benzoyl-20-deoxyingenol; G. Leucodelphinidin; H. Schizonepetoside E; **I.** Melianol; and **J.** Arbutin with the receptor **IXU7**. Melianol showed the highest docking score (−8.475) compared with the reference antidiabetic drugs B. Gliclazide and C. Metformin. The 3D protein binding has been presented with respective amino acid grooves for ligand interaction., Figure S5: Interactions based on **A**. site map of seven ligands B. Gliclazide; C. Metformin; **D**. Picrasonide E; **E.** Azedarachin C; **F.** 3-*o*-Benzoyl-20-deoxyingenol; G. Leucodelphinidin; H. Schizonepetoside E; **I.** Melianol; and **J.** Arbutin with the receptor **2BEL**. Melianol showed the highest docking score (−8.995) compared with the reference antidiabetic drugs B. Gliclazide and C. Metformin. The 3D protein binding has been presented with respective amino acid grooves for ligand interaction, Figure S6: Interactions based on **A**. site map of three ligands **C**. Leucodelphinidin; D. Schizonepetoside E; and **E.** Arbutin with the receptor **GR4F**. Arbutin showed the highest docking score (−7.492) compared with the reference antidiabetic drug B. Metformin. The 3D protein binding has been presented with respective amino acid grooves for ligand interaction., Figure S7: Interactions based on **A**. site map of seven ligands B. Gliclazide; C. Metformin; **D**. Picrasonide E; **E.** Azedarachin C; **F.** 3-*o*-Benzoyl-20-deoxyingenol; G. Leucodelphinidin; H. Schizonepetoside E; **I.** Melianol; and **J.** Arbutin with the receptor **3A5J**. 3-*o*-Benzoyl-20-deoxyingenol showed the highest docking score (−6.123) compared with the reference antidiabetic drugs B. Gliclazide and C. Metformin. The 3D protein binding has been presented with respective amino acid grooves for ligand interaction.

## Sample Availability

Samples of the compounds are not available from the authors.

## Abbreviations

HDL	High-density lipoprotein
LDL	Low-density lipoprotein
TC	Total cholesterol
TFC	Total flavonoid content
TG	Triglyceride
TPC	Total phenolic content
UPLC-QTOF	Ultra-performance liquid chromatography coupled with time-of-flight mass spectrometry
