# Supplementary material for: Pharmacoinformatics and UPLC-QTOF/ESI-MS-Based Phytochemical Screening of Combretum indicum against Oxidative Stress and Alloxan-Induced Diabetes in Long–Evans Rats
Source: Molecules. 2021 Jul 30;26(15):4634. doi: 10.3390/molecules26154634 (PMC8348006; doi:10.3390/molecules26154634)
Supplement: Supplementary file 1 [file molecules-26-04634-s001.zip › molecules-1273178-supplementary.pdf]

Pharmacoinformatics and UPLC-QTOF/ESI-MS-Based Phytochemical Screening of *Combretum indicum* against Oxidative Stress and Alloxan-Induced Diabetes in Long–Evans Rats

Md. Shaekh Forid [1], Md. Atiar Rahman [2,*], Mohd Fadhlizil Fasihi Mohd Aluwi [3], Md. Nazim Uddin [4],
Tapashi Ghosh Roy [5], Milon Chandra Mohanta [6], AKM Moyeenul Huq [7,*] and Zainul Amiruddin Zakaria [8,*]

[1] Department of Pharmacy, School of Science and Engineering, Southeast University, Dhaka 1213, Bangladesh; foridpharmacy91@gmail.com
[2] Department of Biochemistry & Molecular Biology, University of Chittagong, Chittagong 4331, Bangladesh
[3] Faculty of Industrial Science and Technology, Universiti Malaysia Pahang, Pahang 26300, Malaysia; fasihi@ump.edu.my
[4] Institute of Food Science and Technology, Bangladesh Council of Scientific and Industrial Research, Dhaka 1205, Bangladesh; nazimbio@yahoo.com
[5] Department of Chemistry, University of Chittagong, Chittagong-4331, Bangladesh; tapashir57@cu.ac.bd
[6] Department of Chemistry, School of Science and Engineering, Tulane University, New Orleans, LA 70118, USA; milonmohanta@yahoo.com
[7] Department of Pharmacy, School of Medicine, University of Asia Pacific, 74/A, Green Road, Dhaka-1205, Bangladesh
[8] Department of Biomedical Science, Faculty of Medicine and Health Sciences, Universiti Putra Malaysia, UPM Serdang 43400 Selangor, Malaysia
* Correspondence: atiar@cu.ac.bd (M.A.R.); moyeenul.rph@uap-bd.edu (A.K.M.M.H.); zaz@upm.edu.my (Z.A.Z.); Tel.: +88-0312606001-14 (M.A.R.); +88-01906790224 (A.K.M.M.H.); +6019-2117090 (Z.A.Z.)

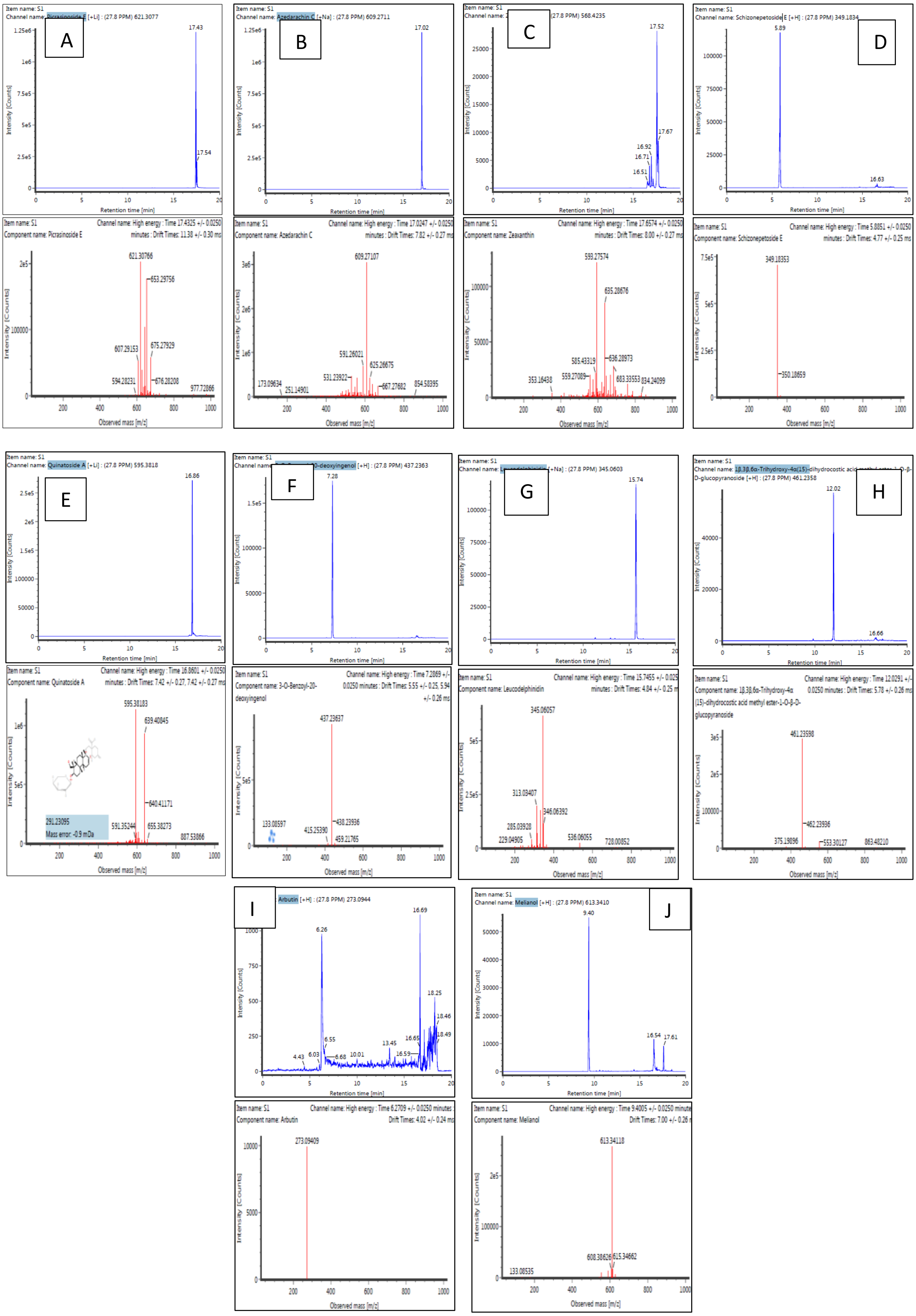

Figure S1: LC chromatogram and mass spectra of CILEx for high-intensity compounds identified and vertically arranged as (**A**) Picrasinoside E; (**B**) Azedarachin C; (**C**) Zeaxanthin; (**D**) Quinatoside A; (**E**) 3-*O*-Benzoyl-20- deoxyingenol; (**F**) Leucodelphinidin; (**G**) Schizonepetoside E; (**H**) 1β,3β,6α-Trihydroxy-4α(15)-dihydrocostic acid methyl ester-1-*O*-β-Dglucopyranoside; (**I**) Melianol; (**J**) Arbutin.

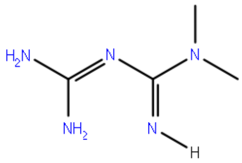

Metformin

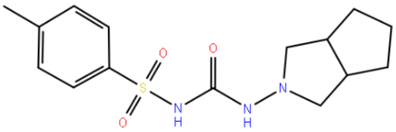

Gliclazide

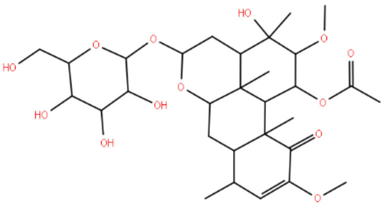

Picrasonide_E

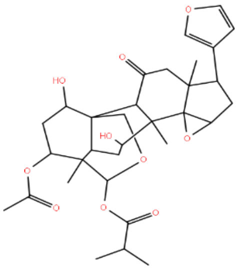

Azedarachin C

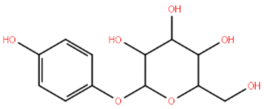

Arbutin

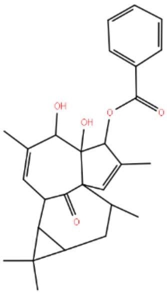

3-O-Benzoyl-2-O-deoxyingenol

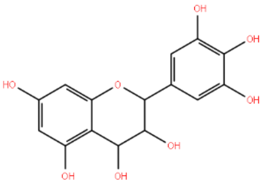

Leucodelphinidin

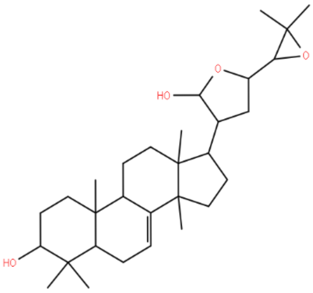

Melianol

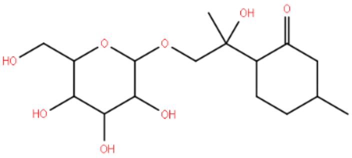

Schizonepetoside E

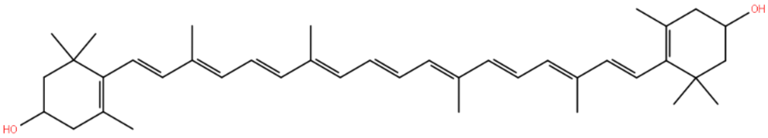

Zeaxanthin

Figure S2: list of chemical structures of the ligand molecules available in PubChem.

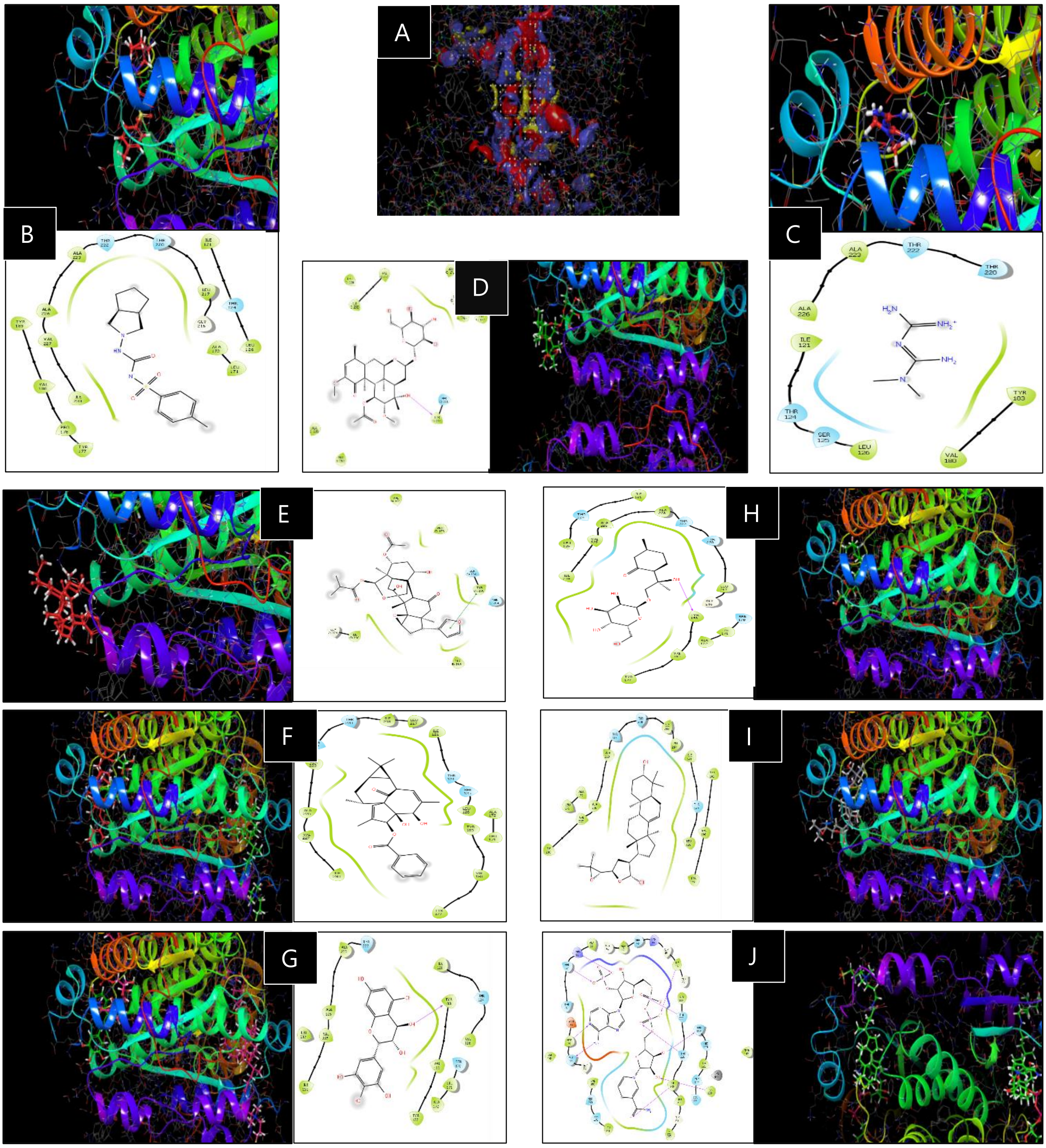

Figure S3: Interactions based on **A**. site map of seven ligands **D**. Picrasonide E; **E.** Azedarachin C; **F.** 3-*O*-Benzoyl-20-deoxyingenol; **G.** Leucodelphinidin; H. Schizonepetoside E; **I.** Melianol; and **J.** Arbutin with the receptor **IXU9**. Schizonepetosie showed the highest docking score (−8.145) compared with the reference antidiabetic drugs B. Gliclazide and C. Metformin.

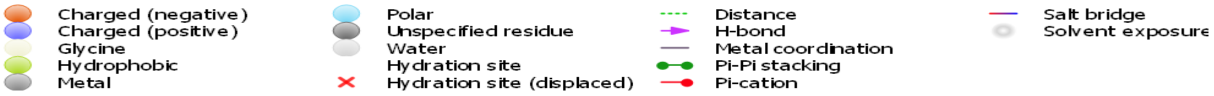

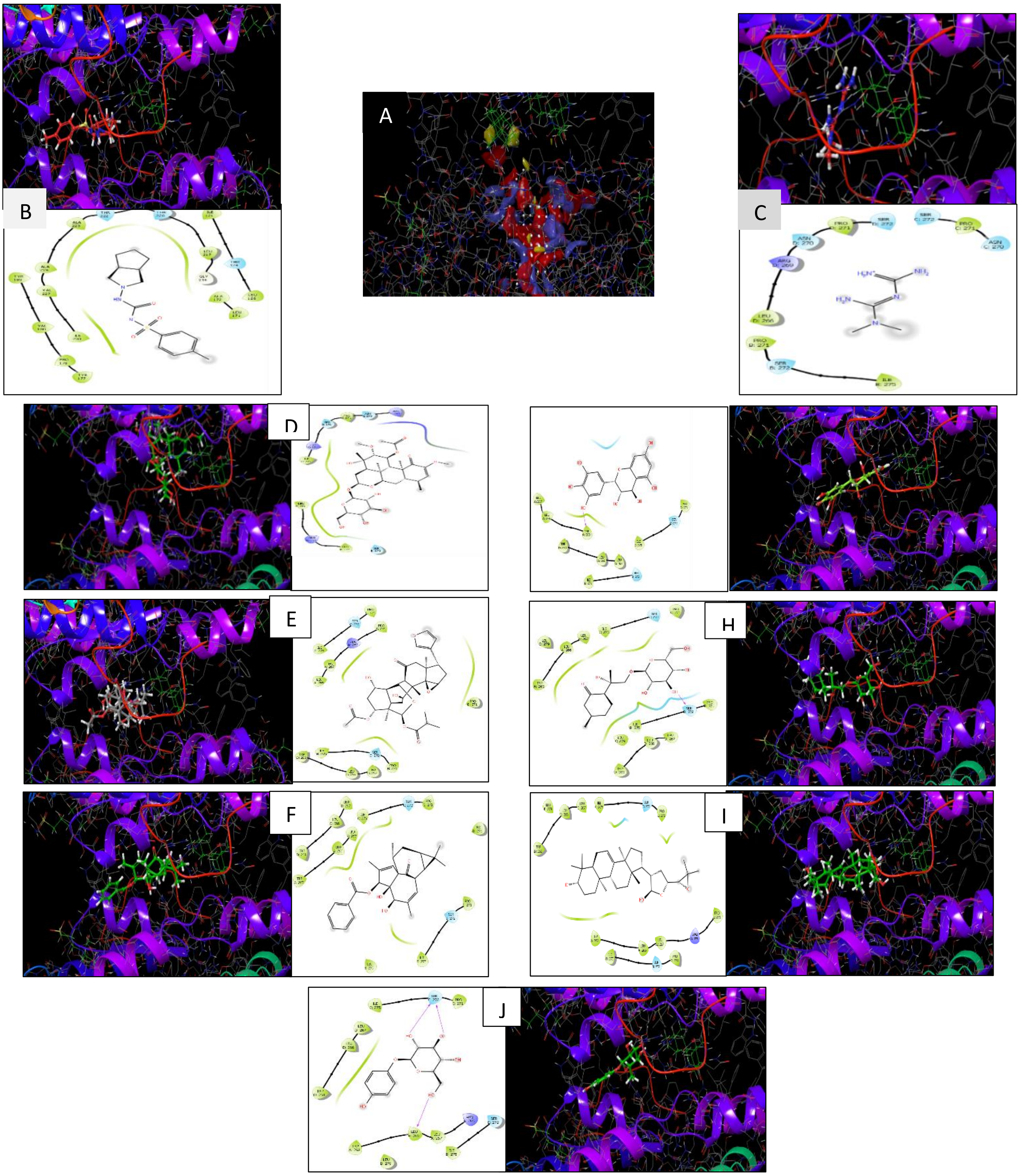

Figure S4: Interactions based on **A**. site map of seven ligands **D**. Picrasonide_E; **E.** Azedarachin C; **F.** 3-*O*-Benzoyl-20-deoxyingenol; G. Leucodelphinidin; H. Schizonepetoside E; **I.** Melianol; and **J.** Arbutin with the receptor **IXU7**. Melianol showed the highest docking score (−8.475) compared with the reference antidiabetic drugs B. Gliclazide and C. Metformin. The 3D protein binding has been presented with respective amino acid grooves for ligand interaction.

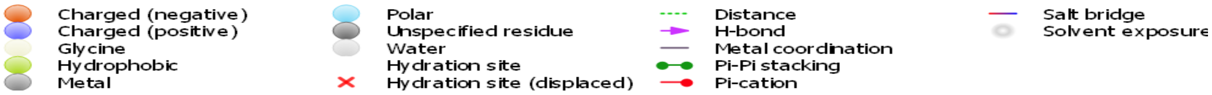

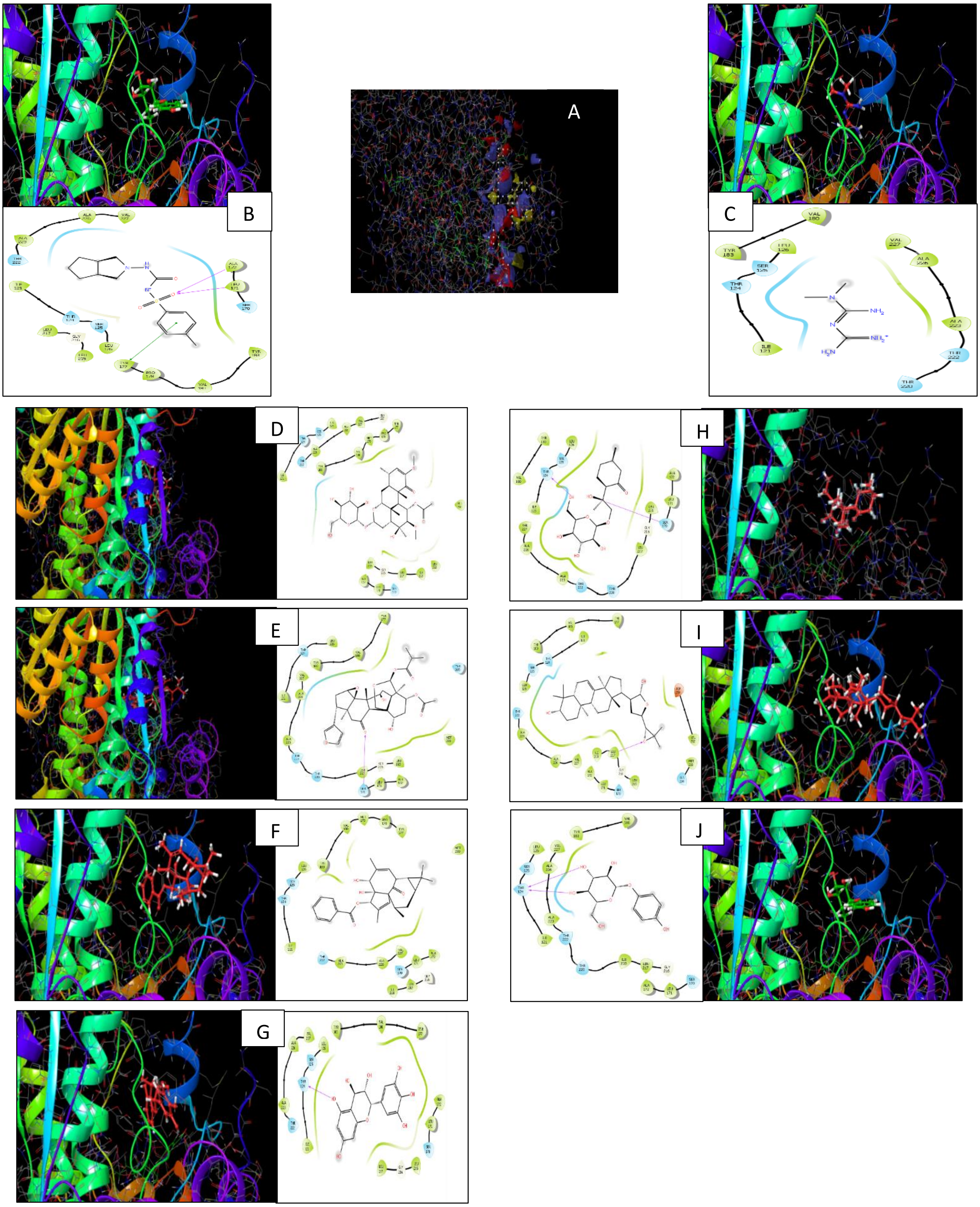

Figure S5: Interactions based on **A**. site map of seven ligands B. Gliclazide; C. Metformin; **D**. Picrasonide E; **E.** Azedarachin C; **F.** 3-*O*-Benzoyl-20-deoxyingenol; G. Leucodelphinidin; H. Schizonepetoside E; **I.** Melianol; and **J.** Arbutin with the receptor **2BEL**. Melianol showed the highest docking score (−8.995) compared with the reference antidiabetic drugs B. Gliclazide and C. Metformin. The 3D protein binding has been presented with respective amino acid grooves for ligand interaction.

Charged (negative)
Charged (positive)
Glycine
Hydrophobic
Metal
Polar
Unspecified residue
Water
Hydration site
Hydration site (displaced)
Distance
H-bond
Metal coordination
Pi-Pi stacking
Pi-cation
Salt bridge
Solvent exposure

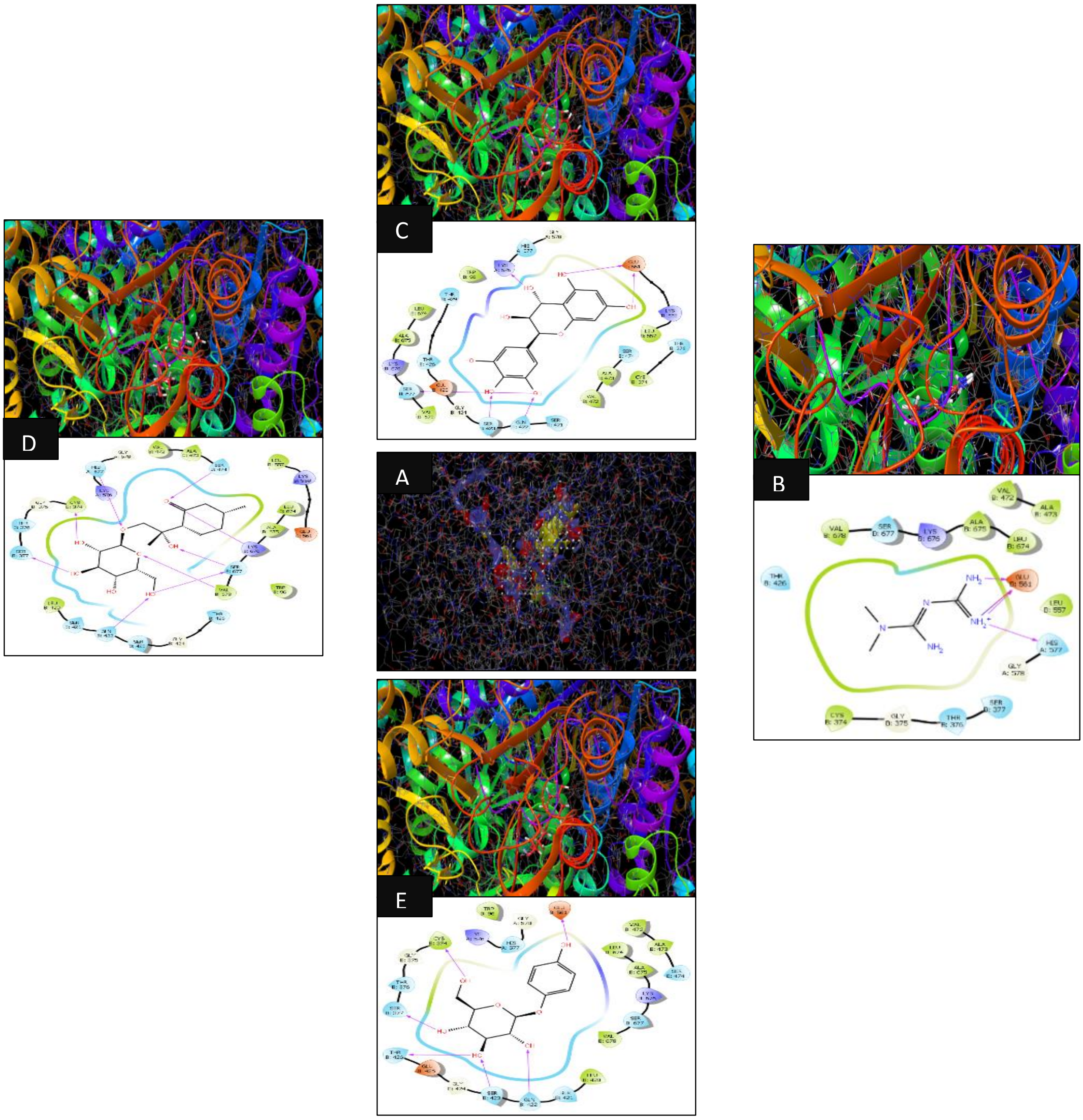

Figure S6: Interactions based on **A**. site map of three ligands **C**. Leucodelphinidin; D. Schizonepetoside E; and **E.** Arbutin with the receptor **GR4F**. Arbutin showed the highest docking score (−7.492) compared with the reference antidiabetic drug B. Metformin. The 3D protein binding has been presented with respective amino acid grooves for ligand interaction.

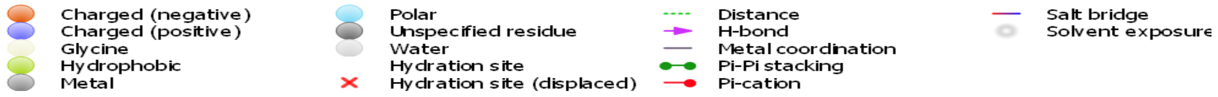

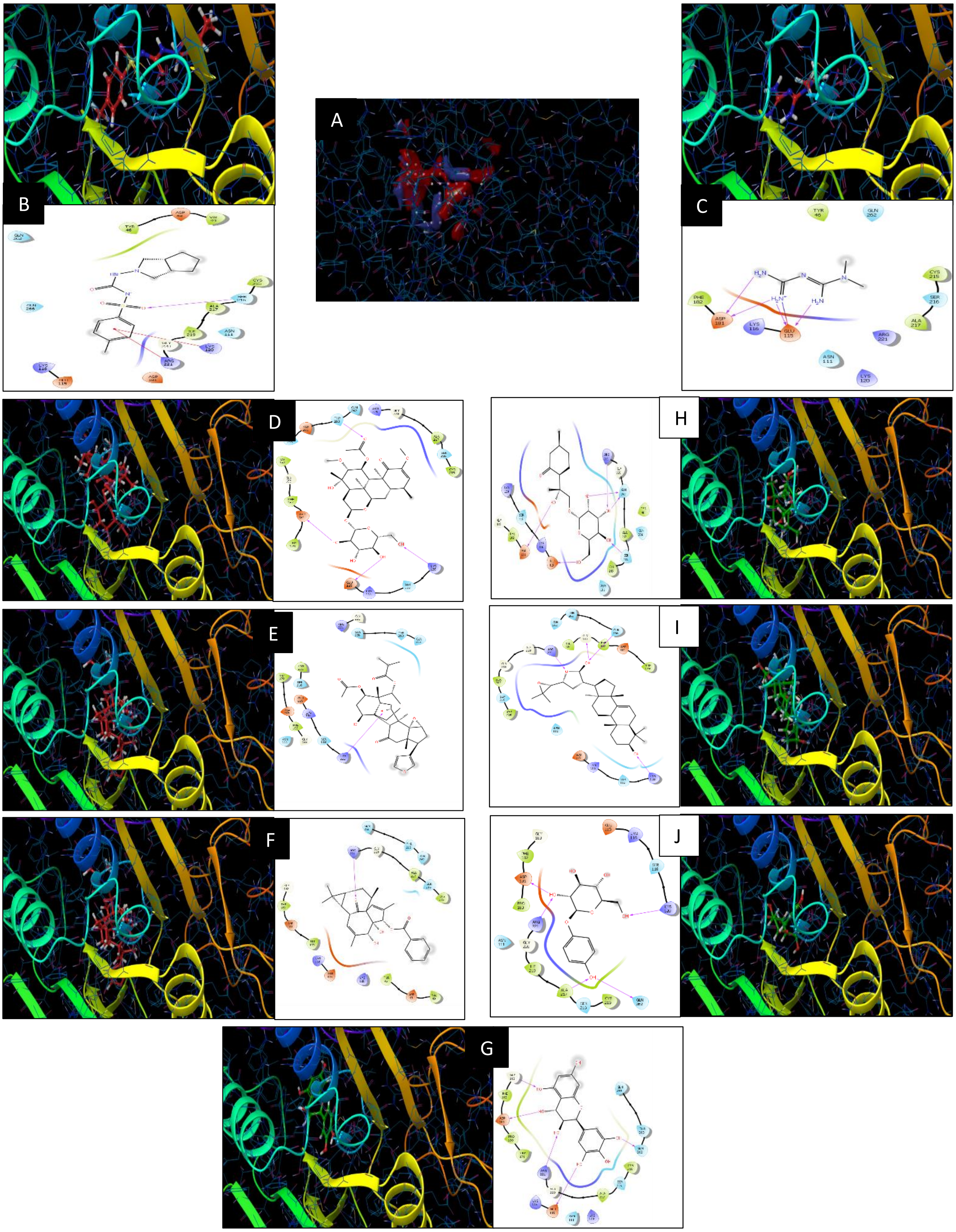

Figure S7: Interactions based on **A**. site map of seven ligands B. Gliclazide; C. Metformin; **D**. Picrasonide E; **E.** Azedarachin C; **F.** 3-*O*-Benzoyl-20-deoxyingenol; G. Leucodelphinidin; H. Schizonepetoside E; **I.** Melianol; and **J.** Arbutin with the receptor **3A5J**. 3-*O*-Benzoyl-20-deoxyingenol showed the highest docking score (−6.123) compared with the reference antidiabetic drugs B. Gliclazide and C. Metformin. The 3D protein binding has been presented with respective amino acid grooves for ligand interaction.

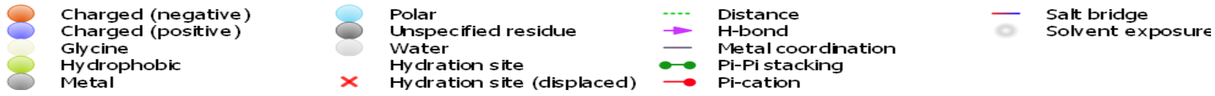
Charged (negative)
Charged (positive)
Glycine
Hydrophobic
Metal
Polar
Unspecified residue
Water
Hydration site
Hydration site (displaced)
Distance
H-bond
Metal coordination
Pi-Pi stacking
Pi-cation
Salt bridge
Solvent exposure
